# Engineered and decellularized human cartilage graft exhibits intrinsic immunosuppressive properties and full skeletal repair capacity

**DOI:** 10.1073/pnas.2507185123

**Published:** 2026-01-09

**Authors:** Alejandro Garcia Garcia, Sujeethkumar Prithiviraj, Deepak Bushan Raina, Tobias Schmidt, Sara Gonzalez Anton, Laura Rabanal Cajal, David Hidalgo Gil, Magnus Tägil, Axel Hyrenius-Wittsten, Madelene W. Dahlgren, Robin Kahn, Paul E. Bourgine

**Affiliations:** ^a^Cell, Tissue and Organ Engineering Laboratory, Department of Clinical Sciences Lund, Lund University, Lund 22362, Sweden; ^b^Wallenberg Centre for Molecular Medicine, Lund Stem Cell Centre, Lund University Cancer Centre, Lund University, Lund 22362, Sweden; ^c^The Faculty of Medicine, Department of Clinical Sciences Lund, Division of Orthopaedics, Lund 22362, Sweden; ^d^Wallenberg Centre for Molecular Medicine, Lund University, Lund 22362, Sweden; ^e^Department of Rheumatology, Institute of Clinical Sciences Lund, Lund University, Lund 22362, Sweden; ^f^Division of Clinical Genetics, Department of Laboratory Medicine, Lund University, Lund 22362, Sweden; ^g^Division of Molecular Hematology, Department of Laboratory Medicine, Lund University, Lund 22362, Sweden; ^h^Department of Pediatrics, Institute of Clinical Sciences Lund, Lund University, Lund 22362, Sweden

**Keywords:** tissue engineering, endochondral ossification, decellularization, extracellular matrices, immune response

## Abstract

Tissue engineering strategies predominantly consist of the autologous generation of living substitutes capable of restoring damaged body parts. Persisting challenges with patient-specific approaches include inconsistent performance, high costs, and delayed graft availability. Toward developing a one-for-all solution, a more attractive paradigm lies in the exploitation of dedicated cell lines for the fabrication of human tissue grafts. Following decellularization, this new class of biomaterials relies on the sole extracellular matrix and embedded growth factors instructing endogenous repair. This conceptual approach was previously validated using a custom mesenchymal cell line for the manufacturing of human cartilage, exhibiting remarkable osteoinductive capacity following lyophilization. Key missing criteria to envision clinical translation include proper decellularization as well as stringent assessment of both immunogenicity and regenerative performance. Here, we report the engineering and subsequent decellularization of human cartilage tissue with minimal matrix impairment. Ectopic evaluation in immunocompetent (IC) and immunocompromised animals reveals preservation of osteoinductivity predicted by macrophage kinetic of polarization. By establishing in vitro human allogeneic coculture models, we evidenced the immunosuppressive properties of cell-free human cartilages, controlling macrophage and dendritic cell maturation as well as T cell activation. Finally, regenerative performance was stringently assessed in an IC rat orthotopic model whereby decellularized human cartilage grafts achieved morphological and mechanical restoration of all critical-sized femoral defects. Taken together, our study provides robust safety and efficacy prerequisites prompting a first-in-human trial for engineered and decellularized human tissue grafts.

## Significance

Tissue engineering creates living substitutes to repair damaged body parts. Patient-specific methods can be costly, slow, and unreliable. A better approach uses special cell lines to produce tissue grafts. After removing the cells, the remaining structure and growth signals help the body heal naturally. This method was tested using a custom cell line to create human cartilage, which showed strong bone-healing ability. For clinical translation, key challenges include proper cell removal, reducing immune reactions, and proving effectiveness. Our study successfully engineered and removed cells from human cartilage while keeping its healing properties. In rats, these grafts repaired bone defects, showing their potential for safe and effective use in future human trials.

Musculoskeletal traumas remain the first cause of disability affecting over 1.7 billion people worldwide (1). Bone injuries and disorders account for a large fraction of this burden (2), creating a substantial demand for effective treatments that an aging population only renders more acute.

Tissue engineering has led to the development of a myriad of biomaterials and bioengineered substitutes shown to stimulate bone regeneration (3). To enhance regenerative performance, a significant breakthrough involved incorporating developmental engineering principles into design strategies. This approach harnesses the natural process of endochondral ossification, where cartilage tissue is engineered to act as a transient template for bone formation (4). To this end, Hypertrophic cartilage (HyC) can be generated in vitro by differentiating various cell sources, such as primary human mesenchymal stem/stromal cells (hMSCs) on scaffolding materials (5). Upon implantation, the engineered HyC is gradually remodeled into mature bone matrix through the differentiation of chondrocytes into osteoblasts and the recruitment of endogenous osteoprogenitor cells (6). Despite showing great promise in terms of graft integration and bone tissue restoration, key challenges inherent to autologous approaches remain to be overcome. These include donor site morbidity, limited availability and variability, as well as inconsistencies in protocols for differentiating hMSCs into chondrocytes (7, 8). These factors add layers of complexity, making it difficult to standardize and optimize the process for consistent and effective clinical outcomes.

Limits of living strategies steered the development of an acellular alternative, whereby the regenerative process would solely rely on the graft extracellular matrix and embedded factors. Foundational studies progressively provided evidence that human engineered cartilage can retain osteoinductive properties despite the absence of a living fraction (9, 10). Robust decellularization techniques were further introduced to remove cellular debris from engineered grafts, increasing the safety of resulting materials (11). Yet, published strategies still rely on the performance of primary human cells for the formation of a mature cartilage template, maintaining concerns about consistency and performance predictability.

Our group has made significant contributions to this conceptual approach by engineering cartilage tissues in vitro using the mesenchymal sword of damocles (MSOD)-B line as a source of immortalized hMSCs (12). We demonstrated the scalable production of human cartilage that retains strong osteoinductive properties even after lyophilization. This work established the foundation for manufacturing human tissue substitutes as off-the-shelf biomaterials capable of guiding and promoting bone repair.

While providing a strong proof-of-concept, the journey to clinical translation remains challenging due to the lack of complete decellularization and stringent performance assessments. The preclinical validation of cell-based but cell-free grafts is further complicated by the fact that this new class of biomaterials is yet to be fully defined by regulatory agencies (1, 13). Additionally, the suitability of standard preclinical models remains to be determined, as often falling short on replicating the complexity of the human immune system and physiological responses (14, 15).

In this study, we aim at achieving the generation of D-Hyc and at determining both its immunogenicity and bone-repair capacity. We first propose to identify an effective decellularization protocol preserving the engineered tissue, followed by the evaluation of the resulting osteoinductive properties and immune recruitment using immunodeficient (ID) and immunocompetent (IC) mouse models. We further design in vitro human immunogenicity assays, in which allogeneic antigen-presenting cells are analyzed for their ability to activate an immune response following exposure to HyC. Finally, grafts will be tested as a callus replacement biomaterial in a critical-sized femoral defect model. By providing both safety and efficacy data, our work aims at prompting the clinical translation of cell line–derived human tissue as grafts engineered to instruct endogenous repair.

## Results

### Cell Line Engineered Human Cartilage Can Be Efficiently Decellularized with Minimal Matrix Impairment.

**Fig. 1.**
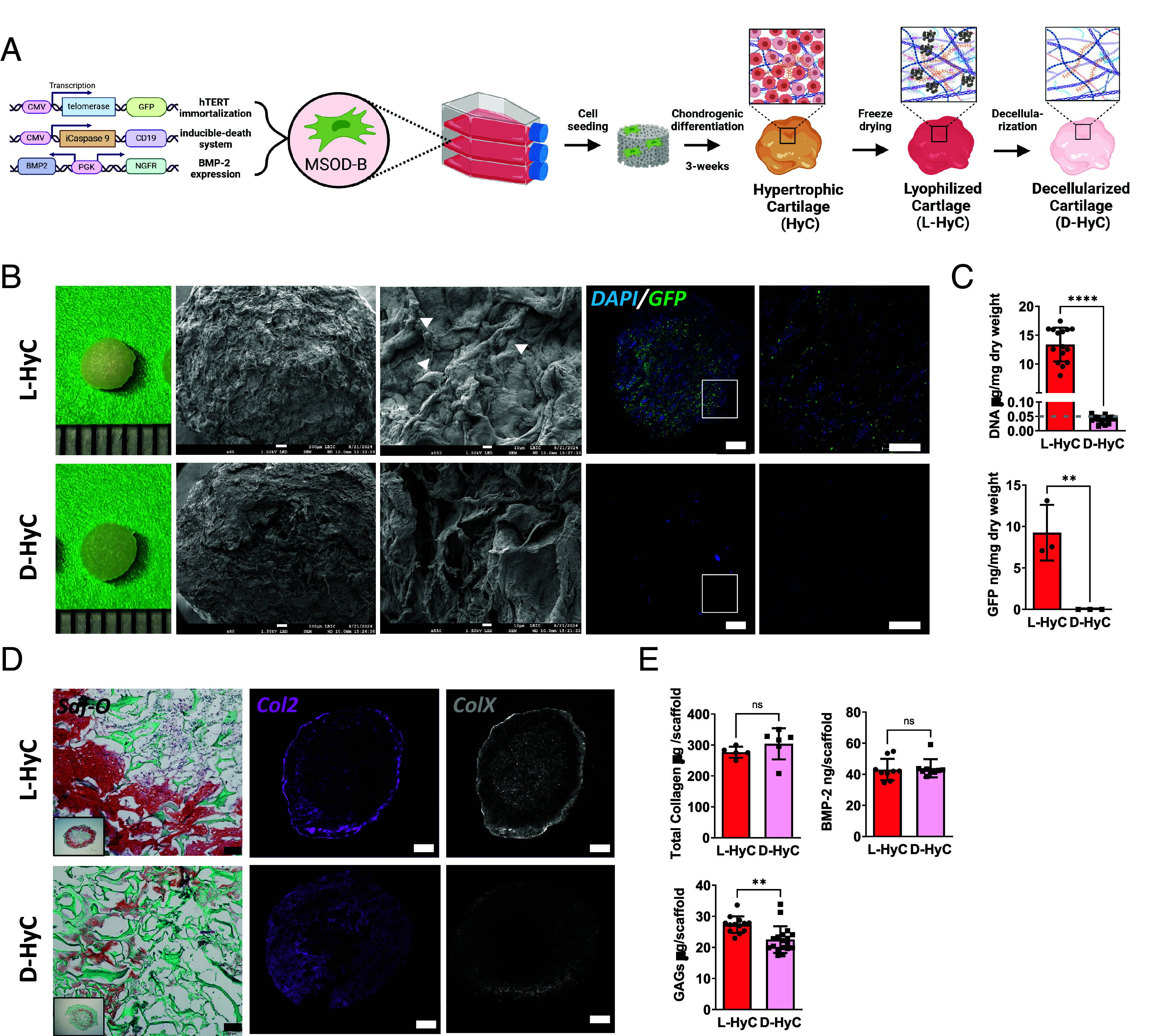
Cell line engineered human cartilage can be efficiently decellularized with minimal matrix impairment. (*A*) Experimental scheme for the generation of decellularized human cartilage grafts. (*B*) From left to right: Macroscopic representation; scanning electron microscopy pictures of graft surfaces (arrows indicate cells). Scale bar, 1 mm and 100 μm; and immunofluorescence confocal images of grafts stained for DAPI and GFP. (*C*) From top to bottom: quantification of total DNA (n = 15) and GFP (n = 3) remnants per mg dry weight of constructs. The dashed line represents the reported threshold for efficient decellularization (0.05 μgDNA/mg dry weight) established in the literature (*D*) From left to right: Safranin-O staining and immunofluorescence images of human grafts stained for Col2 and ColX. (Scale bar, 1 mm.) (*E*) Quantification of GAGs (n = 15), total collagen (n = 6), and BMP-2 content (n = 9) on decellularized and lyophilized grafts. Graphs represent mean ± SD, **P ≤ 0.01, ***P ≤ 0.001, determined by the two-tailed unpaired *t* test.

To identify an optimal decellularization protocol offering both efficient DNA removal and preservation of tissue integrity, 12 protocols were evaluated including distinct detergent treatments (Triton X-100, sodium deoxycholate, and sodium dodecyl sulfate (SDS)), hypertonic and hypotonic baths and DNase digestion time (*SI Appendix*, Fig. S1*A*). Human HyC constructs were in vitro engineered as previously described (12) using the MSOD-B line (Fig. 1*A*), an immortalized hMSCs capable of chondrogenesis. After lyophilization, tissues were exposed to various decellularization protocols and readouts consisted in measurements of total DNA and total glycosaminoglycans (GAGs) amount for decellularization efficiency and matrix preservation, respectively. Three protocols (Protocols 6, 9, and 12) were selected based on their reduced impact on GAGs content and efficient DNA removal and further evaluated by assessing BMP-2 and total collagen preservation (*SI Appendix*, Fig. S1*B*), as well as histological analysis (*SI Appendix*, Fig. S1*C*) of the resulting cartilage constructs. This allowed the identification of one protocol demonstrating efficient decellularization with good preservation of graft constituents (Protocol 12, *SI Appendix*, Fig. S1*A*). The resulting decellularized HyC (D-Hyc) was further analyzed and directly compared to its nondecellularized but lyophilized counterpart (L-HyC). Scanning electron microscopy radiographs revealed an increase surface porosity and exposure of the fibrillar matrix existing within the D-HyC tissue, with no cells visible on the surface (Fig. 1*B*). Taking advantage of the MSOD-B cell GFP expression (Fig. 1*A*), confocal imaging of cartilage tissues revealed a homogenous cellular distribution in L-HyC. Instead, D-HyC staining evidenced an absence of both GFP and DAPI (nuclei) signals, confirming the decellularization efficacy. Quantification of both DNA and GFP levels in digested tissues corroborated the successful decellularization, with D-HyC samples exhibiting negligible DNA levels (0.039 µg/mg of dry weight) as compared to L-HyC (13.4 µg/mg of dry weight). This corresponded to a >99.7% decellularization efficiency (Fig. 1*C*), and residual DNA traces were below the suggested threshold defined in the field (16). To quantify the impact on the cartilage ECM, we further assessed histologically the presence of GAGs, collagen type-2 and collagen type-X (Fig. 1*D*). While both L-HyC and D-HyC display features of HyC matrix, the D-HyC samples showed a noticeable GAGs reduction and a disrupted collagen-2/collagen-X networks as compared to L-HyC. This was further confirmed by a quantitative Blyscan assay, revealing a 17.6% loss in GAG content compared to nondecellularized controls (Fig. 1*E*). In contrast, analysis of other structural proteins indicated the preservation of total collagens composition (Fig. 1*E*). Similarly, the levels of BMP-2 embedded within the matrix remained unaffected (Fig. 1*E*).

We further characterized the reproducibility in L-HyC and D-HyC production by assessing potential batch-to-batch variations. By measuring DNA, GAGs, and BMP-2 content across batches (*SI Appendix*, Fig. S2*A*), we demonstrated a high reproducibility in cartilage tissue quality both pre- and postdecellularization. Endotoxin levels were additionally shown to be below the suggested Food and Drug Administration threshold for medical device (*SI Appendix*, Fig. S2*A*), falling in the range of clinically available collagen scaffolds (Avitene™ Ultrafoam™). Last, we evaluated in vitro the retention of biological activity of our human cartilage matrices. To this end, L-HyC and D-HyC were grinded into a fine-powder toward supplementation in culture media. Primary hMSCs were cultured in standard complete medium supplemented or not with the cartilage matrices powder, to assess an impact on the cell proliferation (mitogenic assessment) or osteogenic differentiation (osteoinductive assessment). The supplementation of L-HyC/D-HyC powder had no effect on the primary hMSCs proliferation (*SI Appendix*, Fig. S2*C*). Instead, we observed a priming of primary hMSCs toward osteogenic differentiation upon powder supplementation, as evidenced by the deposition of a calcified matrix (*SI Appendix*, Fig. S2*D*), although not reaching the amount obtained using an osteogenic medium (positive control, *SI Appendix*, Fig. S2*D*). These data evidence that L-HyC and D-HyC retained some biological activities, primarily in the form of osteoinductive capacity.

Taken together, we here report the identification of an efficient decellularization protocol, affecting GAGs content but preserving collagens and BMP-2 compositions. By exploiting the MSOD-B line, we could achieve the production of D-HyC displaying minimal batch-to-batch variations and preserved biological activity.

### Decellularized Human Cartilage Grafts Fully Remodeled into Bone Organs by Ectopic Priming of Endochondral Ossification in ID Mice.

**Fig. 2.**
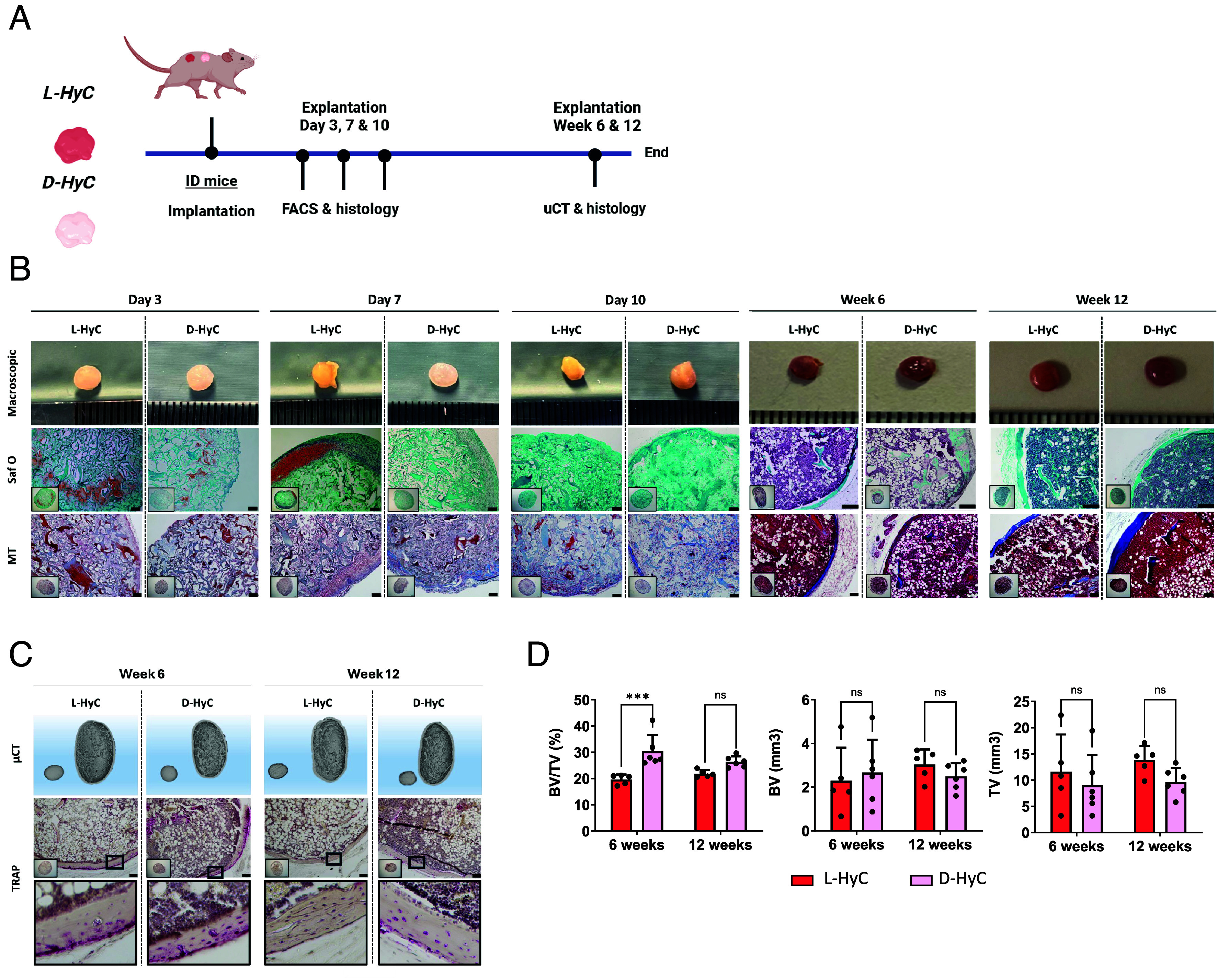
Decellularized human cartilage grafts fully remodeled into bone organs by ectopic priming of endochondral ossification in ID mice. (*A*) Experimental scheme for the in vivo ectopic osteogenic assessment of L-HyC and D-HyC grafts. (*B*) From top to bottom, representative macroscopic images (Scale bar, 1 mm), histological images of Safranin-O and Masson Trichrome staining of explanted tissues. (Scale bar, 100 µm.) (*C*) From top to bottom, representative µCT 3D reconstruction and TRAP staining of explanted tissues at 6 and 12 wk. (Scale bar, 400 µm.) *Bottom* images represent a higher magnification of the region selected in the upper images (black square). (*D*) BV/TV (%), BV (mm^3^), and TV (mm^3^) of µCT performed over explanted bones at 6 and 12 wk postimplantation (n ≥ 5). The graphs represent mean ± SD, ***P ≤ 0.001, determined by two-way ANOVA.

To assess whether the decellularization process affects the capacity of human engineered cartilage grafts to prime endochondral ossification, we implanted L-HyC and D-HyC grafts ectopically into ID BALB/C nude mice (Fig. 2*A*). Early time-point analysis revealed similar remodeling of L-HyC and D-HyC, with progressive cell colonization. By day 10, the cartilage matrix has been fully degraded by host cells (Safranin-O) accompanied by persistence of a collagen matrix, as confirmed by Masson’s trichrome staining (MT) (Fig. 2*B*).

Remarkably, by 6 wk postimplantation both L-HyC and D-HyC grafts fully remodeled into mature bone and bone marrow tissues (Fig. 2*B*). Safranin-O and Masson’s trichrome stainings demonstrated the replacement of cartilage with mature bone tissue, including marrow infiltration and the formation of a cortical bone layer with trabecular structures (Fig. 2*B*). Micro-CT analyses confirmed macroscopical observations while TRAP staining revealed active osteoclast-mediated remodeling at 6 wk, consistent with mature bone turnover (Fig. 2*C*). At 12 wk postimplantation, tissues persist in vivo marking a definite remodeling with no detectable osteoclastic activity. Micro-CT radiographs allowed quantification of bone mineralization (Fig. 2*D*) and tissue volume at 6 and 12 wk postimplantation. This confirmed the similar performance of both L-HyC and D-HyC tissues, with only a superior bone volume/tissue volume ratio (BV/TV) in D-HyC at the 6 wk timepoint. By 12 wk, no significant differences in both bone and tissue volume were found between groups.

As control, we also implanted the pure collagen scaffold (Avitene™ Ultrafoam™) which serves as the starting material for in vitro cartilage production through MSOD-B seeding and differentiation. After 6 wk of implantation, all collagen scaffolds were fully resorbed and failed to generate bone (*SI Appendix*, Fig. S3*A*). In contrast, we confirmed robust bone and bone marrow formation in additional ID models (*SI Appendix*, Fig. S3*B*).

These results demonstrate the strong osteoinductive properties of engineered human cartilage grafts and evidence the intrinsic bone forming capacity of fully decellularized human cartilage tissue.

### Decellularized Human Cartilage Does Not Induce Robust Ectopic Bone Formation in IC Mice.

Given the efficient decellularization achieved, we aimed to evaluate whether our grafts could similarly achieve ectopic bone formation when exposed to a fully functional immune system. To this end, L-HyC and D-HyC tissues were implanted into C57BL/6 and tissue remodeling was assessed across various time-points (*SI Appendix*, Fig. S4*A*). Within the first 10 d postimplantation, the cartilage matrix is progressively degraded in both L-HyC and D-HyC (*SI Appendix*, Fig. S4*B*). Concomitantly, a thick collagen layer was observable at the periphery of the implants, suggesting an encapsulation.

At later time points (6 and 12 wk), none of the retrieved samples exhibited frank bone formation histologically (*SI Appendix*, Fig. S4*B*), mostly consisting of fibrous collagen matrix. While D-HyC and L-HyC exhibited similar histological features, their retrieval rates largely differed. Most L-HyC samples were fully degraded by the host, with only 40% and 20% of tissues being recovered at 6 and 12 wk, respectively. In turn, 80% of D-HyC were found at both 6 and 12 wk postimplantation. Among retrieved samples, TRAP stainings fail at detecting any bone remodeling but small areas of mineralization could be identified in D-HyC samples only (*SI Appendix*, Fig. S4 *C* and *D*).

These findings highlight the immunogenic nature of the human matrix components within engineered cartilage grafts, leading to rejection at ectopic site in IC mice. The removal of human cells and DNA suggest a reduction of the immune reaction associated with higher persistence of D-HyC tissues, although failing at priming endochondral ossification.

### Immune Prints of Engineered Human Cartilage Correlate Early M0 to M2 Polarization with Successful Ectopic Bone Formation.

Given the successful bone formation observed in ID and failure in IC animals, we seeked to further investigate the underlying inflammatory properties of L-HyC and D-HyC grafts in both ID and IC mice. To do so, we performed a temporal analysis of immune recruitment in the first days (3, 7, and 10) postimplantation, corresponding to the reported acute inflammatory phase preceding remodeling (17, 18).

**Fig. 3.**
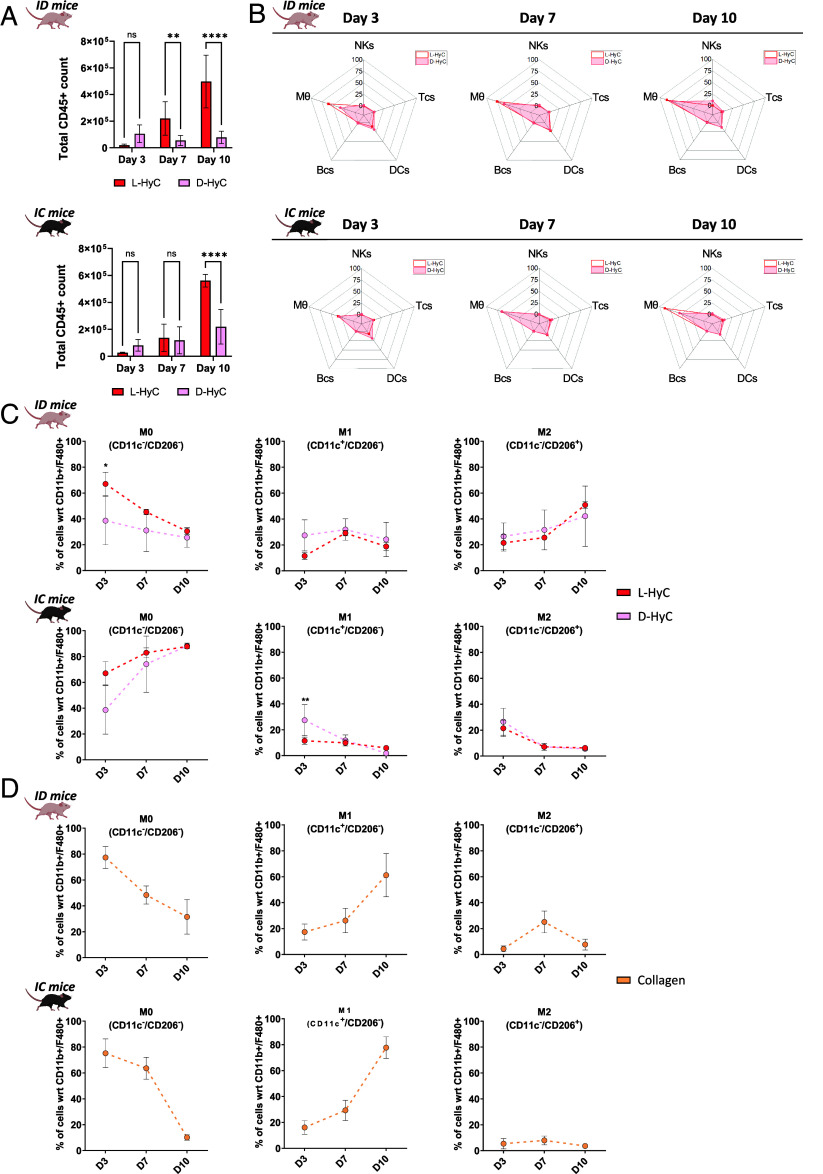
Immune prints of engineered human cartilage correlate to early M0 to M2 polarization with successful ectopic bone formation. (*A*) Total cell number captured in explanted tissues after 3, 7, and 10 d postimplantation in ID (*Top*) and IC (*Bottom*) animals. The graphs represent mean ± SD, **P ≤ 0.01, ****P ≤ 0.0001, determined by two-way ANOVA. (*B*) From right to left, representative radar plots representing percentages of early time recruitment of B cells, T cells, dendritic cells, natural killers, and macrophages captured in explanted tissues after 3, 7, and 10 d, respectively, in ID (*Top*) and IC (*Bottom*) animals. (*C*) Percentage of CD11b^+^ and F4-80^+^ macrophage subtypes in L-HyC and D-HyC grafts explanted at 3, 7, and 10 d postimplantation analyzed by flow cytometry in ID (*Top*) and IC (*Bottom*) animals. The graphs represent mean ± SD, *P ≤ 0.1, ***P ≤ 0.001, determined by two-way ANOVA. N ≥ 3 independent experiments, 3 pooled samples per animal (n ≥ 9). (*D*) Percentage of CD11b^+^ and F4-80^+^ macrophage subtypes in explanted collagen scaffolds after 3, 7, and 10 d, respectively, in ID (*Top*) and IC (*Bottom*) animals. N ≥ 3 independent experiments, 3 pooled samples per animal (n ≥ 9). The graphs represent mean ± SD.

The total number of cells recruited to implants was shown to gradually increase over time (*SI Appendix*, Fig. S5*A*), oscillating between 0.5 and 1 M cells per graft in both ID and IC settings. In ID animals, a consistent superior recruitment was observed in L-HyC as compared to D-HyC. A similar trend was observed in IC with L-HyC recruiting more cells, although not reaching significance. This difference in cell recruitment largely accounted for blood/immune cell infiltration (CD45+, Fig. 3*A*), particularly marked at days 7 and 10 postimplantation. To further identify and discriminate the different recruited immune populations, a comprehensive panel of phenotypic markers was set for flow cytometry analysis (*SI Appendix*, Fig. S5*B*). This allowed compiling “immune prints,” capturing the immune recruitment landscape in implanted grafts as a snapshot in respective timepoints and settings (Fig. 3*B* and *SI Appendix*, Fig. S5*C*). These radar plots consistently identified macrophages (MΦ), natural killer cells (NKs), dendritic cells (DCs), T cells (Tcs), and B cells (Bcs) within the implanted tissues. Strikingly, the immune prints of L-HyC and D-HyC revealed to be very similar across all timepoints as well as between ID and IC animals. Macrophages were identified as the most abundant recruited cell type, with their frequency peaking on day 10 postimplantation. While both grafts’ immune prints closely aligned, D-HyC exhibited a statistically lower macrophages frequency at day 3 and increased NKs at day 10 in ID animals. In IC, D-HyC exhibited statistically higher DCs at day 3, higher T cell frequency at day 7 and day 10 and increased NK at day 10. On the other hand, they exhibited lower frequency of macrophages at day 10. (*SI Appendix*, Fig. S5*C*).

As a control, we also implanted collagen scaffolds to comprehend the immune recruitment induced by the material deprived of any cartilage matrix. Similarly to L-HyC and D-HyC, macrophages were abundantly recruited in the scaffold (*SI Appendix*, Fig. S5*D*). Their percentage gradually increases over time, reaching up to 70% of CD45+ cells. No major differences between IC and ID recruitment were observed in collagen scaffolds, besides higher DCs in early timepoints in IC.

Based on the observed predominant presence of macrophages in all constructs, we further explored their distinct polarization states (Fig. 3*C*). Importantly, macrophage polarization dynamics were similar between L-HyC and D-HyC grafts. However, substantial pattern differences could be observed by comparing ID and IC animals. In ID, we observed an initial dominant unpolarized (M0) state which progressively declined overtime. Concomitantly, a progressive increase in proregenerative macrophages (M2) was observed reaching its peak by day 10. Instead, the proinflammatory M1 population remains stable, suggesting a M0 to M2 polarization process. Instead, in IC animals we report a progressive increase in M0 macrophages, reaching over 88% of total macrophages by Day 10 (Fig. 3*C*). In sharp contrast with the ID animals, no increase in M1 nor M2 populations was observed, those populations even declining over the 10 d observational course.

Instead, collagen scaffolds displayed very identical patterns in both ID and IC animals (Fig. 3*D*). A striking and progressive polarization into M1 was observed, concomitant with a decrease in unpolarized M0. The presence of M2 macrophages remained low, despite a peak observed at Day 7 in the ID setting.

Taken together, the early immune profiling of L-HyC and D-HyC exhibit comparable inflammation which aligns with the similar osteoinductive performance in ID and IC. By further correlating immune profile and bone formation, our data suggest that a M0-to-M2 polarization is required and predictive of successful ectopic ossification, as observed for both L-HyC and D-HyC. This is reinforced by the collagen control data, whereby no M0-to-M2 pattern can be associated with resorption of the tissue in vivo (6 wk). The L-HyC and D-HyC immune prints suggest a similar immunogenicity of our grafts despite the decellularization. Alternatively, it may evidence limits of in vivo models in assessing immune reaction caused by human grafts.

### Human Cartilage Grafts Induce T Cell Engagement but Low CD8 Recruitment in the Nur-GFP Mouse Model.

To further comprehend whether our engineered grafts elicit an adaptive immune response in vivo, we employed the Nur77-GFP reporter mice. This transgenic reporter model is based on the Nur77 (Nr4a1) promoter driving GFP expression (19), thereby providing a readout of T cell receptor (TCR) engagement and signaling (*SI Appendix*, Fig. S6*A*). D-HyC and L-Hyc grafts were implanted subcutaneously in Nur77-GFP animals as previously described and explanted 10 d later for phenotypic analysis of the T cell immune compartment. As a control, collagen scaffolds were similarly implanted and analyzed.

The percentage of CD3+ T cells recruited to the grafts (*SI Appendix*, Fig. S6*B*) was similar across groups, although collagen scaffold showed a trend for higher infiltration (21.65 ± 13.55, 8.54 ± 5.44 and 7.08 ± 3.04% for Collagen, L-HyC, and D-HyC, respectively). Absolute CD3^+^ T cell counts did not differ significantly among conditions. Importantly, all groups contained a substantial fraction of TCR-engaged T cells (CD3+GFP+, *SI Appendix*, Fig. S6*B*), with 43.60 ± 25.98, 52.68 ± 19.16 and 54.95 ± 16.51% for Collagen, L-HyC, and D-HyC groups, respectively.

Further analysis of T cell subsets revealed a comparable proportion of CD4+ T cells in all conditions (*SI Appendix*, Fig. S6*C*). However, collagen scaffolds exhibited a significantly higher infiltration of CD8+ T cells (26.75 ± 17.30%, *SI Appendix*, Fig. S6*C*), representing a fivefold increase compared to L-HyC (5.26 ± 1.48%) and D-HyC (6.57 ± 2.13%) grafts. The IFN-γ and IL-2 cytokines—two canonical markers of T cell activation- were detected at low levels in explanted grafts but showed no statistical differences between groups (*SI Appendix*, Fig. S6*D*).

Together, these findings demonstrate that implantation of engineered human cartilage grafts in IC mice engages the adaptive immune system, as evidenced by TCR activity in a substantial portion of recruited CD3^+^ T cells. Notably, engineered cartilage grafts displayed immunogenicity comparable to the clinically approved collagen scaffold, while eliciting a markedly lower CD8^+^ T cell response.

### Human Allogeneic In Vitro Assays Reveal Minimal Macrophage Polarization and Absence of T Cell Activation by Decellularized Human Cartilage Grafts.

**Fig. 4.**
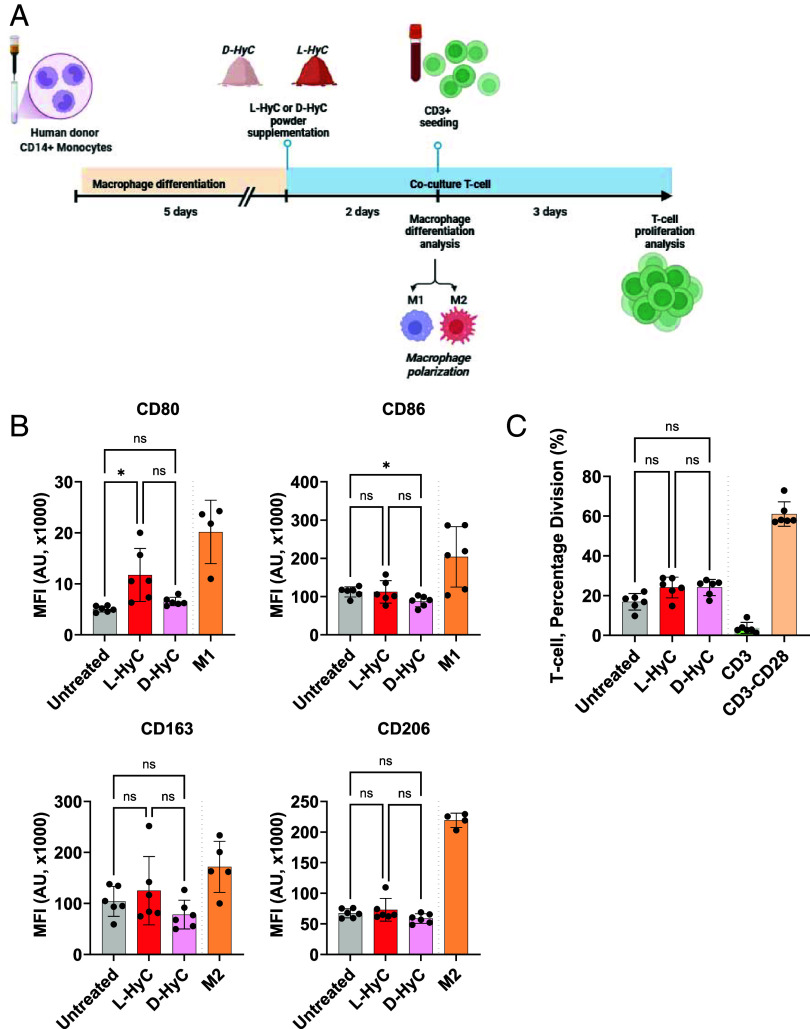
Human allogeneic in vitro assays reveal minimal macrophage polarization and absence of T cell activation by decellularized human cartilage grafts. (*A*) Experimental scheme of the direct effect of powdered L-HyC and D-HyC on macrophage polarization and indirect T cell activation potential. (*B*) From top to bottom, total CD80+ and CD86+ MFI indicating M1 polarization; and total CD163+ and CD206+ MFI indicating M2 polarization, after 5 d coculture with either L-HyC or D-HyC powdered cartilages. The graphs represent mean ± SD, *P ≤ 0.1 determined by repeated measures one-way ANOVA (6 donors, n = 3 per donor). (*C*) CD3+ percentage division assessed by FACS after coculture of 5 × 10^4^ CD3+ cells with 2.5 × 10^3^ macrophages cocultured with either L-HyC or D-HyC powdered cartilages. The graphs represent mean ± SD, determined repeated measures one-way ANOVA, statistical significance set at *P* < 0.05 (6 donors, n = 3 per donor).

Toward mimicking an inflammation scenario upon clinical implantation, we investigated the immunogenicity of engineered cartilage grafts in a fully human setting by design of in vitro assays. To this end, we first developed a setup whereby monocytes from healthy individuals (n = 6 donors) were isolated and differentiated toward macrophages (Fig. 4*A*). Their polarization status was assessed postdifferentiation, when exposed to L-HyC or D-HyC. As positive polarization controls, macrophages were exposed to LPS and IFN-γ to form M1 macrophages, or IL-10 and dexamethasone for M2 macrophages induction. To maximize cell exposure to the graft material, both L-HyC and D-HyC were cryomilled into a fine powder and subsequently added to the culture medium. After macrophage differentiation, flow cytometry revealed an increase in proinflammatory M1 markers (CD80 and CD86) upon exposure to L-HyC (Fig. 4*B*). Instead, D-HyC did not induce a significant M1 polarization, as compared to macrophages not exposed to any HyC materials (Untreated). Moreover, none of the grafts were shown to induce M2 polarization (CD163 and CD206, Fig. 4*B*). Comparing polarization patterns across donors (*SI Appendix*, Fig. S7 *A* and *B*), we observed variable M1 activation in the L-HyC group, whereas D-HyC consistently led to lower activation of proinflammatory macrophage markers. Similarly, while L-HyC generally induced a stronger M2 response compared to D-HyC, this effect was highly donor-dependent.

Next, we evaluated the capacity of resulting macrophages to differently induce T cell activation/proliferation after exposure to L-HyC or D-HyC (Fig. 4*C*). As a positive control, T cells were treated with anti-CD3 and anti-CD28, where CD28 acts as costimulatory signal leading to full TCR activation. Anti-CD3 only was used to directly stimulate TCR, leading to incomplete activation. After 3 d, only the CD3-CD28 group exhibited substantial T cell proliferation (60%). In contrast, T cells cocultured with macrophages exposed to either L-HyC or D-HyC showed minimal activation (Fig. 4*C*). These results indicate that macrophages exposed to L-HyC or D-HyC, despite an increase of costimulatory molecules following L-HyC stimulation, do not significantly induce T cell proliferation. It further reveals that DNA and cellular remnants in L-HyC did not cause an increased inflammatory macrophage-driven response. Furthermore, the absence of significant differences between untreated and construct-exposed groups suggests that neither L-HyC nor D-HyC per se are capable of triggering T cell activation. In fact, coculturing T cells with M1 or M2 macrophages (*SI Appendix*, Fig. S7*C*) resulted in the same level of T cell activation as observed with L-HyC and D-HyC tissues. This suggests that the engineered grafts do not further amplify macrophage-driven inflammation beyond the baseline macrophage activation state. Instead, T cell activation is primarily driven by CD3 signaling rather than macrophage phenotype and associated inflammatory cytokines. In fact, our findings indicate that HyC matrices do not push macrophages toward a highly inflammatory M1-like state nor enhance their antigen presentation capacity. Last, we tested the capacity of grinded collagen scaffolds to induce T cell proliferation in a similar assay. This revealed that collagen induces a higher CD3 T cell proliferation than D-HyC (*SI Appendix*, Fig. S7*D*).

Overall, our data revealed that the decellularization of human cartilage grafts reduced M1 polarization while minimizing donor-to-donor variability. Unlike findings from mouse studies, no M2 polarization was observed in our in vitro assays. Notably, engineered L-HyC and D-HyC materials minimized T cell activation induced by macrophages.

### Human Engineered Cartilage Grafts Do Not Elicit a Human Adaptive Immune Response In Vitro while Exhibiting Immunosuppressive Properties.

**Fig. 5.**
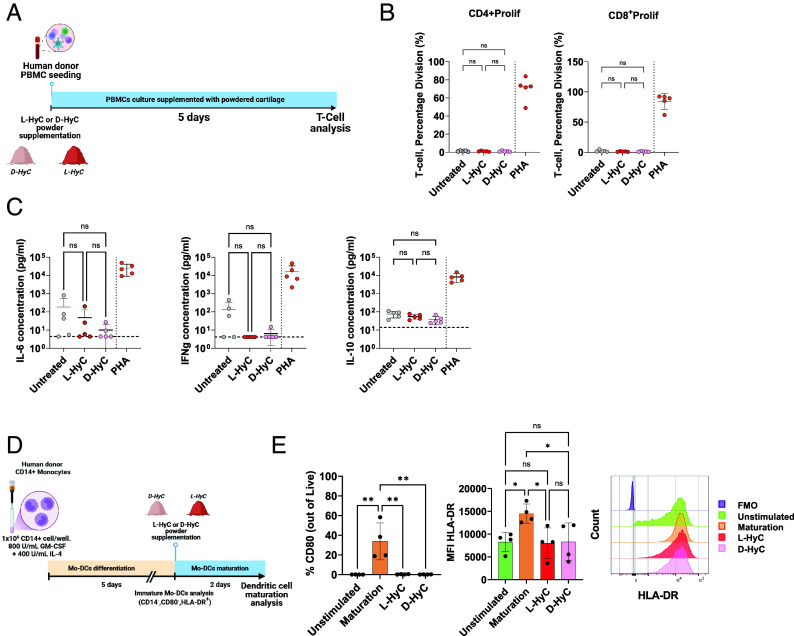
Human engineered cartilage grafts do not elicit a human adaptive immune response in vitro while exhibiting immunosuppressive properties. (*A*) Experimental scheme of the direct effect of powdered L-HyC and D-HyC on T cell activation when directly cocultured with PBMCs for 5 d. (*B*) From right to left, percentage of activated CD3+CD4+ and CD3+CD8+ T cells. The graphs represent mean ± SD, determined by repeated measures one-way ANOVA, statistical significance set at *P* < 0.05. (*C*) Proinflammatory cytokines released by PBMCs after 5 d coculture with either L-HyC or D-HyC powdered cartilages. The graphs represent mean ± SD, determined by repeated measures one-way ANOVA, statistical significance set at *P* < 0.05. The horizontal dashed lines indicate the detection threshold of the ELISA. (*D*) Experimental scheme of the assessment of dendritic cell maturation when cultured with either L-HyC or D-HyC powdered cartilages or maturation media in combination with powdered cartilages for 48H. (*E*) From left to right, percentage of CD80+ and total HLA-DR+ MFI of dendritic cells when exposed with either L-HyC, D-HyC powdered cartilages or maturation media in combination with powdered cartilages for 48H. The graphs represent mean ± SD, **P ≤ 0.01, determined by ordinary one-way ANOVA.

In light of the observed macrophages response, we further assessed whether an adaptive immune response could be mounted after exposure to L-HyC or D-HyC. We thus isolated full mononuclear cells (PBMCs) -containing T cells- and similarly exposed them to grinded human cartilages for 5 d (Fig. 5*A*). Phytohemagglutinin (PHA) was used as a positive control ensuring T cell proliferation, whereas PBMCs not exposed to any human cartilage was set as a control group (Untreated).

As anticipated, the PHA condition strongly induces T cell proliferation with 74% and 87% of CD4 or CD8 T cells, respectively entering division (Fig. 5*B*). In stark contrast, PBMCs exposed to the human cartilage epitopes were unable to activate T cells (Fig. 5*B*). Strikingly, both CD4+ and CD8+ did not enter proliferation, with a percentage of T cell division similar to the untreated group (Fig. 5*B*). Inflammatory cytokines (IFN-γ, IL-6, and IL-10) measured in the coculture medium confirmed those observations (Fig. 5*C*), with L-HyC and D-HyC values equivalent to those from the Untreated group. Taken together, these data confirmed the low immunogenicity induced by L-HyC and D-HyC in vitro (Fig. 5*C*).

Using flow cytometry, we further analyzed T cell activation status by assessing subtype distribution based on surface marker expression. As anticipated, the PHA group exhibited the largest activation of various T cell subsets (*SI Appendix*, Fig. S8*A*). However, no differences across HyC groups could be observed, with a detected but limited percentage of CD4^+^CD25^+^, CD4^+^HLA-DR^+^, CD8^+^CD25^+^ and CD8^+^HLA- DR^+^ T cell subsets (*SI Appendix*, Fig. S8*A*). This suggests an immunosuppressive effect of HyC regardless of their decellularization status.

The absence of T cell activation questioned whether the human cartilage grafts can impair the function of antigen-presenting cells. As DCs account for professional antigen-presenting entity, we assessed whether their maturation could be affected by the L-HyC or D-HyC matrices. To this end, monocytes were first isolated from healthy donors and predifferentiated into immature DCs (Fig. 5*D*). At that stage, over 99% of the cells expressed typical DC markers (CD14^−^HLA-DR^+^) but not activated ones (<1% of CD80+) (*SI Appendix*, Fig. S8*B*). Immature DCs were then exposed to a maturation media (positive control) or to the L-HyC and D-HyC grinded proteins to assess their capacity to induce DCs maturation. The maturation media led to a robust DCs activation with 33% of cells expressing the CD80 markers, in conjunction with an increase in mean HLA-DR expression intensity (Fig. 5*E*). In contrast, when exposed to L-HyC or D-HyC, immature DCs fail at further differentiating, with no detectable CD80 expression and no increase in HLA-DR intensity (Fig. 5*E*).

In summary, those findings evidenced the low immunogenic nature of cell-free human cartilage grafts. Using in vitro human-based assays, the decellularization process did not significantly reduce the inflammatory status as L-HyC and D-HyC presented similar-to-identical-properties. Interestingly, despite the absence of living cells, the cartilage ECMs displayed immunoregulatory features linked with reduced T cell activation by PBMCs and impairment of DC maturation.

### D-Hyc Promotes Full Healing of Critical-Sized Femoral Defect in IC Rats.

**Fig. 6.**
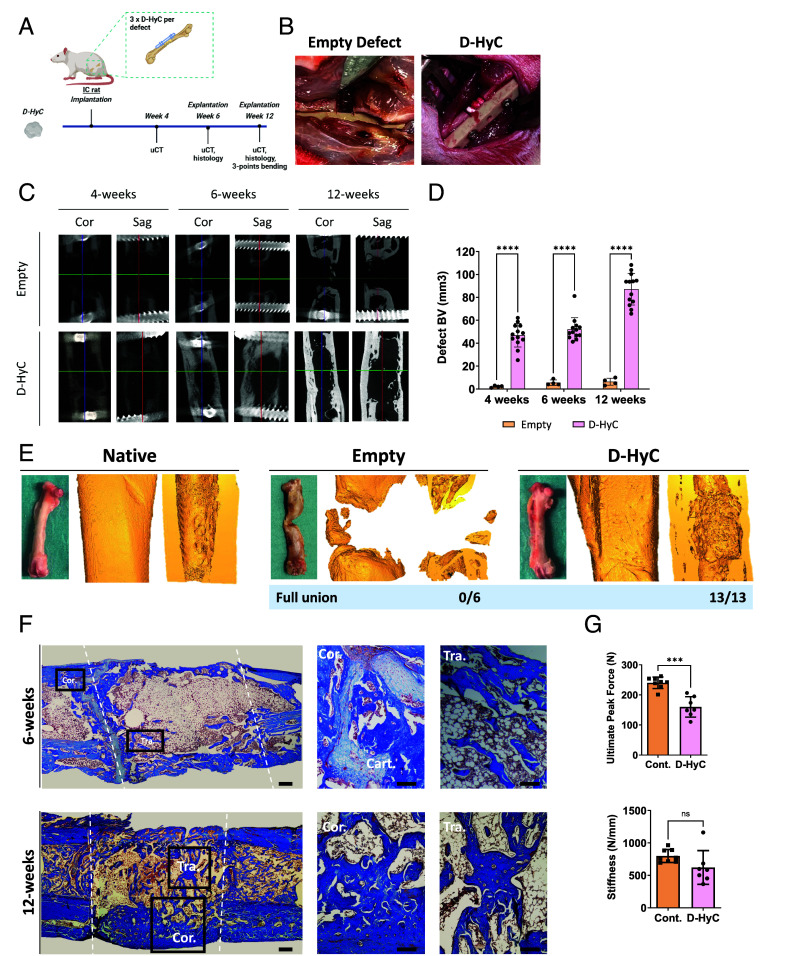
D-Hyc promotes full healing of critical-sized femoral defect in IC rats. (*A*) Experimental scheme of the assessment of bone regenerative potential of decellularized tissue engineered cartilages assessed in a critical full-sized femoral defect in IC rats. (*B*) From left to right, macroscopic images of the empty critical full-sized defect and the defect filled with a total of three engineered HyC tissues. (*C*) Micro-CT-based representative 2D coronal (*Left*) and sagittal (*Right*) views from the middle of the defect after 4, 6, and 12 wk for empty groups and regenerated femurs implanted with D-HyC. (*D*) Quantification of micro-CT-based BV in the defect (region of interest height = 4.5 mm) for empty groups and regenerated femurs implanted with D-HyC after 4, 6, and 12 wk of implantation. The graphs represent mean ± SD, ****P ≤ 0.0001, determined by two-way ANOVA. (*E*) From left to right, representative macroscopic image of an explanted regenerated femur at 12 wk; representative 3D images of the entire defect area in both whole and cross-sectional view. (*F*) From left to right, representative histological images of Masson Trichrome staining of explanted femurs completely regenerated after 12 wk implantation with D-HyC; and a magnification of a region of interest of a cortical (cor.) and a trabecular (tra.) bone structure. (Scale bar, 500 and 100 µm, respectively.) (*G*) From left to right, Ultimate Peak Force (N) and total Stiffness (N/mm) for nonoperated contralateral bones and regenerated femurs implanted with D-HyC after 12 wk of implantation (n = 7). The graphs represent mean ± SD, ***P ≤ 0.001, determined by two-way ANOVA.

After demonstrating potent osteoinductivity and reduced immunogenicity, we last assessed the repair capacity of our human engineered graft in a preclinically relevant setting. To this end, D-HyC tissues were implanted into a critical-sized femoral defect in an IC rat model (Fig. 6*A*). To fill the 5 mm defect, 3-constructs were implanted at the surgical site (Fig. 6*B*), with the graft diameter approximating the diameter of the femur. As negative control, defects were left untreated (empty controls). The repair capacity was monitored for up to 12 wk by micro-computed tomography (micro-CT) followed by histological assessments.

As early as 4 wk, micro-CT revealed significant mineralization in D-HyC implants as compared to empty controls (Fig. 6*C*). Quantification across timepoints confirmed the progressive repair, with potential defect bridging visualized at 6 wk in 12 out of 13 animals (Fig. 6 *C* and *D*). Bone/mineralized volume in the defect region constantly increased reaching 87 mm^3^ by 12 wk.

Femurs from animals were then explanted at 6 and 12 wk for histological analysis. Remarkably, upon explantation, macroscopic and micro-CT evaluation revealed full-bridging in the D-HyC-treated group (Fig. 6*E*). This was the case for all operated animals (13/13), with D-HyC femurs resembling the nonoperated contralateral ones (*SI Appendix*, Fig. S9). Instead, empty controls did not exhibit robust repair (Fig. 6*E*). 3D reconstructions and cross-sectional views confirmed robust bone regeneration in the D-HyC group only and bridging of the defects (Fig. 6*E*). To confirm frank de novo bone formation, we performed histological analysis using Masson Trichrome staining. At 6 wk postimplantation, an extensive bone formation was observed in the defect area, bridging with the adjacent cortical structures (Fig. 6*F*). At that timepoint, a cartilage layer could be observed at the native bone/defect interface, indicating an ongoing endochondral ossification process. Trabecular structures and restoration of the bone marrow cavity were also evidenced at that timepoint. At 12 wk, a more extensive bone trabecular network was observed, along with a thickening of the cortical structure (Fig. 6*F*). At that stage, no cartilage remnants were observed, suggesting a definite regeneration. We further compared the bridging performance of D-HyC with published grafting strategies evaluated in the same IC rat femoral model (*SI Appendix*, Fig. S10) (11, 20–22). Only a high dose of BMP2 and syngeneic transplantation (rat living cartilage graft) match the performance of D-HyC at 12 wk postimplantation, with consistent defect bridging.

Finally, we performed a biomechanical evaluation of retrieved femurs, toward assessing the extent of their functional restoration. D-HyC-treated femurs 12 wk postimplantation were tested, while nonoperated contralateral ones served as positive controls. Using the ultimate peak force assay, D-HyC femurs were shown to achieve 67% of the contralateral femur resistance to fracture (160N versus 240N, for D-HyC versus control, respectively, Fig. 6*G*). Instead, stiffness measurements indicated no statistical differences between D-HyC and control femurs.

## Discussion

In this study, we report the successful generation of D-Hyc, retaining remarkable osteoinductive properties predicted by early M0 to M2 polarization. Independently from the decellularization process, HyC demonstrated potent immunoregulatory properties, characterized by minimal human allogeneic T cell activation and DC reduced maturation. When implanted in a rat critically sized defect, D-HyC led to full femoral regeneration with morphological and mechanical bone restoration.

The decellularization of engineered tissues presents the attractive opportunity to create off-the-shelf grafting solutions, eliminating the need for immunomatching (23, 24). To achieve an effective removal of immunogenic cell debris, existing protocols often compromise the composition and thus the performance of engineered tissues. This challenge is particularly pronounced in cartilage due to its dense matrix (25). Physical methods often disrupt cellular membranes and nuclei, but it may not completely remove all cell components, while chemical processing can disrupt collagen network and cause significant loss of GAGs and growth factors. A tailored combination of chemical and physical methods is needed for effectively decellularizing such tissues (26). In this study, we successfully established a decellularization protocol using an optimized combination of detergents (SDS), hypertonic/hypotonic buffers, and DNase treatment. SDS is widely used in cartilage decellularization at concentrations ranging from 1 to 2% for 24 to 72 h (27, 28). However, due to its cytotoxic effects, which can compromise the biocompatibility of decellularized grafts (29), we optimized the protocol by reducing SDS concentration to 0.5% and limiting exposure to 12 h. To further improve efficiency while preserving ECM integrity, we implemented a stepwise approach, sequentially applying treatments rather than exposing the tissue to all reagents simultaneously. While hypertonic and hypotonic baths are typically used to lyse cells and precipitate cellular debris, we additionally utilized the hypertonic step to aid in SDS removal, minimizing residual irritative effects. In contrast to previous protocols that applied all reagents at once but achieved only partial decellularization and significant GAG loss (30), our controlled, sequential strategy ensures efficient cell removal while better preserving essential matrix components. This resulted in significant removal of cell-associated materials, with residual DNA levels below the recommended threshold (<50 ng/mg dry weight) (16). The full decellularization process is achieved within 2.5 d, below the average of standard protocols (27, 31, 32) (*SI Appendix*, Fig. S11).

Similar to other methods, we report an impact on the deposited ECM principally in the form of a significant GAGs content reduction (26, 33). Nonetheless, changes did not impair the grafts bone-forming capacity in both stringent ectopic and orthotopic models. These findings highlight the ability of the sole extracellular matrix, independent of living cells, to drive endochondral ossification and support robust bone regeneration.

Despite the proven decellularization, robust safety assessment is essential before considering clinical translation. This is particularly important also in regard to the exploitation of a human cell line for the graft engineering process. Ectopic implantation in ID and IC animals is considered standard assay for teratoma and immunogenicity assessment. The ectopic implantation also presents the advantage of assessing active cell recruitment, as compared to extensive bleeding or inflammation typically resulting from orthotopic surgery. Our method allowed compiling a comprehensive temporal and quantitative analysis of immune recruitment, summarized in the form of immune prints. We evidenced the importance of the early innate invasion, dominated by macrophages recruitment similarly to early phases of bone repair (34). Comparing collected profiles in ID and IC, we could correlate the transition of macrophages from an M0 to M2 phenotype during the early inflammatory phase with subsequent success of ectopic ossification. This shift is consistent with the well-established sequence of immune events, where early proinflammatory responses are gradually replaced by a reparative phase characterized by the resolution of inflammation and tissue remodeling (1, 35, 36). M2 macrophages have been largely described as active promoter of angiogenesis, tissue repair, and associated with proregenerative processes in various tissue contexts including bone formation (1, 37–39). While informative, one limitation of our methodology relates to the simplified binary M1/M2 phenotypic definition, typically challenged by the tissue location and inflammation contexts (40, 41).

The ectopic model further allows comparing the in vivo immunogenicity of HyC pre- and postdecellularization. Surprisingly, the decellularization was shown to mostly affect the extent of recruited immune cells, but not their inflammatory profiles. As such, D-HyC exhibited a reduced blood infiltration as compared to L-HyC, consistent across timepoints and animal models. Beyond immune recruitment, L-HyC and D-HyC displayed very similar immune prints, as well as identical proinflammatory M1 versus proregenerative M2 ratios. Corroborated by the absence of an early M2 polarization, none of the grafts initiated an effective remodeling into bone upon implantation in IC mice. Notably, HyC constructs consistently outperformed a conventional material, collagen scaffolds, in both ID and IC models, demonstrating superior macrophage polarization profiles, indicating a lower immunogenic response. In ID mice, HyC exhibited significantly higher M2 polarization, indicative of a strong regenerative drive, whereas collagen scaffolds showed only minimal M2 response and higher M1 presence. Of note, the apparent immune rejection in the ectopic IC model was not associated with T cell expansion, whose frequency remained stable over time for both L-HyC and D-HyC. However, the Nur77-GFP reporter model revealed that a large fraction of T cells displayed engaged TCRs, and the similar proportions observed between human grafts and collagen scaffolds suggest that the response may not be driven by human epitopes.

Existing studies have assessed the performance of living human cartilage in IC models (20, 42), relying on the immunosuppressive properties of differentiated hMSCs to circumvent immune rejection. However, effective bone formation could not be achieved in this xenogeneic setting. Since our D-HyC grafts are devoid of human cells, our study identifies interspecies variations in ECM proteins as the primary cause of human ectopic HyC rejection. This finding highlights the challenge of using animal models to cojointly evaluate the immunogenicity and osteoinductivity of decellularized human grafts.

To complement ectopic findings, we thus established in vitro assays as simplified but fully human settings mimicking an allogeneic response. While several studies evaluated the immunogenicity of living chondrogenic tissue (43–45) and hMSCs in vitro (46, 47), the inflammatory response induced by human decellularized grafts has not been reported. By exposing HyC to the full spectrum of antigen-presenting cells, we could demonstrate that engineered extracellular matrix do not elicit a strong inflammation. Using multiple healthy donors, PBMCs and macrophages resulted in minimum T cell activation for both CD4 and CD8 subsets, in line with low detection of proinflammatory cytokines. Further to that, our engineered ECM grafts were also shown to impact the in vitro maturation/function of human immune cells. In particular, D-HyC significantly reduced both the M1 (proinflammatory) and M2 (proregenerative) macrophages polarization. Grafts were further demonstrated to affect DCs maturation, as evidenced by low levels of standard activation markers (CD80 and HLA-DR). These findings echo the previous results on chondrogenically differentiated hMSCs failing at inducing T cell activation and DC maturation (44, 45, 48, 49). However, our study extends previous findings by demonstrating that decellularized constructs, devoid of living cells, similarly exhibit comparable immunomodulatory properties. This is unexpected, as implying that our engineered ECM and embedded factors are sufficient to confer and retain potent immunoprivileged features, despite the lyophilization and decellularization processes. Taken together, findings from mouse models and human in vitro assays strongly support the safety and allogeneic exploitation of D-HyC as a bone graft substitute with tolerogenic properties. Although our ECMs do completely suppress the immune response, our findings evidence a clear immunosuppression defined as a decrease in immune response activities.

In a final step, D-HyC grafts were evaluated in a rat orthotopic model featuring a critical-sized femoral defect. Compared to preexisting approaches, the graft demonstrated remarkable performance in terms of both reproducibility and bone repair efficacy. Several studies have explored the endochondral ossification pathway and tested HyC potential in orthotopic settings. To the best of our knowledge, our graft outperforms previous strategies by achieving faster and superior bone formation (6, 11, 20, 50–52). However, implantation of a living rat cartilage graft and high doses of BMP-2 also resulted in comparable healing at 12 wk. Those strategies actually mimic current clinical gold standards, consisting in autologous grafting and BMP-2 delivery. Their limitations are well known, reinforcing the relevance of our D-HyC, approach superior in safety and performance. Most importantly, unlike prior studies relying on living implants, our D-HyC is strictly off-the-shelf, and its regenerative performance was assessed without any additional materials. Beyond femoral bridging, mechanical restoration was also observed, although it did not reach the strength of native femurs. We hypothesize that the stabilizing plate may have reduced load-bearing and mechano-stimulation in the defect leg, thereby preventing full restoration of native mechanical properties.

Importantly, in sharp contrast to ectopic observations, the rat IC orthotopic environment did not lead to graft rejection. This highlights that beyond graft composition, the local environment plays a pivotal role in regenerative decision-making (53–55). A limitation of our work consists in the absence of immune populations characterization at the femoral site. This is challenged by the surgical procedure and associated bleeding but would inform on key immune recruitment differences between ectopic and orthotopic sites. More generally, the discrepancy across animal models and site of implantations challenges the establishment of standard criteria to evaluate safety and efficacy of human decellularized biomaterials. The successful clinical translation of our approach will further require overcoming cost of goods and regulatory hurdles, as well as demonstrating efficacy and biosafety in large animal models. From a biosafety and sterility perspective, the production will involve GMP-certified facilities with established sterility and safety testing before release. Critical parameters for regulatory approval include the absence of cell nuclei and minimal levels of residual DNA and detergents, which are standard criteria for approval of decellularized tissues. To comprehensively evaluate efficacy in large animal models, experimental designs should directly assess the D-HyC osteoinductive potential in clinically relevant scenarios. Those include femoral segmental defects in sheep or minipigs, or spinal fusion models in which HyCs can be incorporated within a structural cage or scaffold (e.g., PEEK or metallic cages) to assess their ability to induce vertebral fusion. Cost of goods represents a more complex challenge largely shared by tissue-engineered approaches. Our research-grade production distributes expenses at approximately 70% reagents and 30% personnel. Importantly, larger batch production will effectively reduce the costs of reagents while the automation of specific cell culture processes will in turn increase safety and diminish personnel costs. Toward this direction, robotic-assisted are now emerging and adaptable to the formation of cartilage micropellets (56, 57). The automatization is a key step toward scalability which the use of a cell line largely facilitates and further supports a cost-effective approach toward robust manufacturing.

The clinical translation of our D-HyC graft may be further hindered by the lack of regulatory uniformity, as its classification straddles the line between cell-based and acellular products. This is particularly the case for products that do not involve a recellularization step with patient cells before implantation, such as our D-HyC. Recent regulatory advancements include the Food and Drug Administration (FDA) approval of an engineered acellular vessel (58), resulting from primary human endothelial cells 3D differentiation and subsequently decellularized (59). This product falls in the Biologics definition of the FDA and benefited from the Regenerative Medicine Advanced Therapy designation. This regulatory pathway could similarly apply to D-HyC, given its nature as a cell-based but cell-free product. In contrast, the European Medicines Agency (EMA) lacks a clear equivalent designation, as no such product has ever been released in Europe. Early discussions with EMA representatives suggest that D-HyC would likely be classified as an Advanced Therapy Medicinal Product–Tissue Engineered Product, although this category has so far been applied exclusively to living substitutes. Importantly, although D-HyC originates from a human immortalized cell line, regulators have not regarded this origin as fundamentally distinct from other decellularized tissues. Instead, the product will be evaluated according to the same safety criteria than standard decellularized tissues. This similarity reinforces the feasibility of a regulatory path for cell line–derived decellularized products.

A clear and uniform classification will only facilitate the journey to clinical translation, together with defining preclinical models and in vitro systems modeling the complexity of the human immune system and physiological responses (15, 60). This remains an open challenge, with performance in humans not being predictable particularly regarding their safety, integration, and longevity within the body.

## Conclusion

The development of decellularized tissue engineered grafts represents a promising approach for tissue regeneration, offering a scalable, off-the-shelf solution that bypasses the limitations of autologous and living grafts (23, 61). Using a dedicated human cell line, our work demonstrates the possibility to engineer D-HyC as bone graft substitute exhibiting remarkable bone formation capacity together with intrinsic immunoregulatory functions. Additionally, it underscores the limitations of current in vitro and in vivo models in accurately assessing the immunogenicity and performance of decellularized human grafts.

## Materials and Methods

MSOD-B cells were expanded and seeded on type I collagen sponges to generate cartilage constructs under chondrogenic conditions for 3 wk. Hypertrophic grafts were lyophilized or decellularized and further processed into powders for biochemical analysis and functional testing. In vitro, graft-derived materials were evaluated for their effects on hMSC proliferation, osteogenic differentiation, and immune modulation using monocyte polarization, T cell proliferation, PBMC activation, and dendritic cell maturation assays. For in vivo assessment, grafts were implanted subcutaneously in ID and IC mice to study immune recruitment, tissue remodeling, and ectopic bone formation. Finally, a rat critical-sized femoral defect model was used to test the bone regenerative capacity of decellularized grafts by micro-CT, histology, and mechanical analysis.

## Supplementary Material

Appendix 01 (PDF)

## Data Availability

Study data are included in the article and/or *SI Appendix*. We confirm that the data will be made available upon publication of the article (62).
