# Supplementary material for: Engineered and decellularized human cartilage graft exhibits intrinsic immunosuppressive properties and full skeletal repair capacity
Source: Proc Natl Acad Sci U S A. 2026 Jan 9;123(2):e2507185123. doi: 10.1073/pnas.2507185123 (PMC12799180; doi:10.1073/pnas.2507185123)
Supplement: Supplementary file 1 — Appendix 01 (PDF) [file pnas.2507185123.sapp.pdf]

Supporting Information for

Engineered and decellularized human cartilage graft exhibits intrinsic immuno-suppressive properties and full skeletal repair capacity

Alejandro Garcia Garcia[1,2], Sujeethkumar Prithiviraj[1,2], Deepak Bushan Raina[3], Tobias Schmidt[4,5], Sara Gonzalez Anton[1,2], Laura Rabanal Cajal[1,2], David Hidalgo Gil[1,2], Magnus Tägil[3], Axel Hyrenius-Wittsten[6], Madelene W. Dahlgren[7], Robin Kahn[4,8], Paul Bourgine[1,2,*]

[1]Cell, Tissue & Organ Engineering Laboratory, Department of Clinical Sciences, Lund University, Lund, Sweden

[2]Wallenberg Centre for Molecular Medicine, Lund Stem Cell Centre, Lund University Cancer Centre, Lund University, Lund, Sweden

[3]The Faculty of Medicine, Department of Clinical Sciences Lund, Orthopaedics, Lund, Sweden

[4]Wallenberg Centre for Molecular Medicine, Lund University, Sweden 5Department of Rheumatology, Institute of Clinical Sciences Lund, Lund University, Lund, Sweden

[6]Division of Clinical Genetics, Department of Laboratory Medicine, Lund University, Lund, Sweden

[7]Division of Molecular Hematology, Department of Laboratory Medicine, Lund University, Lund, Sweden

[8]Department of Pediatrics, Institute of Clinical Sciences Lund, Lund University, Lund, Sweden

*Paul.bourgine@med.lu.se

This PDF file includes:

Supporting text
Figures S1 to S11

Supporting Information Text

Extended Materials and Methods

MSOD-B culture. MSOD-B cells (P21) are expanded in T175 flasks in complete medium consisting of alpha Minimum Essential Medium supplemented with 10% fetal bovine serum, 1% HEPES (1M), 1% Sodium pyruvate (100mM), 1% of Penicillin/Streptomycin/Glutamine solution and 5ng/ml of FGFb (all from Gibco Invitrogen, USA) under standard culture conditions until confluence (90%). Medium is replaced twice a week. Cells are seeded at a standard density of 3.400 cells/cm2.

MSOD-B chondrogenic differentiation. MSOD-B cells (P23) are harvested and seeded on cylindrical type I collagen sponges (AviteneTM UltrafoamTM Collagen Sponge, Davol) of 6mm in diameter and 3mm in thick. Briefly, scaffolds are shaped with a 6mm biopsy punch (Kai Biomedical) and placed in a single well of a 12-well plate coated with 1% agarose to prevent cell adhesion. Per scaffold, 35µL of cell suspension containing 2M of cells are seeded at the surface and incubated 1h under standard culture conditions (cell culture media as seen before without FGFb). Then 2mL of chondrogenic medium is added to the well. This medium consisted of DMEM supplemented with 1% Insulin/Transferrin/Selenium, 1% Sodium pyruvate (100mM), 1% of Penicillin/Streptomycin/Glutamine (100X) solution (all from Gibco Invitrogen, USA), 0.47mg/mL linoleic acid (Sigma Aldrich, USA), 25mg/mL bovine serum albumin (Sigma Aldrich A2153-50G), 0,1mM ascorbic acid-2-phosphate, 10ng /ml TGFb3 and 10-7M dexamethasone. Chondrogenic medium is changed twice a week for a period of three weeks.
When indicated throughout the manuscript, collagen sponges of 6mm were exploited as control for *in vivo* and *in vitro* experiments.

Lyophilization. After three weeks of hypertrophic differentiation, MSOD-B constructs are rinsed twice with PBS 7,2 (Gibco Invitrogen, USA). For lyophilization, cell constructs are then snap froze inside 15-ml tubes in liquid nitrogen for 5 minutes and then lyophilized at -80°C and 0.05mbar over night. Once freeze dried, the lyophilized cell constructs (L-HyC) are stored at 4°C for long term storage.

Decellularization. Parameters for the selection and testing of decellularization protocols were selected from pre-existing studies involving detergent-based cellular disruption, osmotic stress, and nuclease treatment(59–61). An iterative approach was adopted to optimize reagent concentrations and incubation times. After lyophilization samples were immersed in a hypertonic solution (Tris-HCl 50mM, NaCl 1.5M, pH 7.6) for 10H at RT over continuous stirring (100rpm). After rinsing 3X with PBS at RT (30min each wash) each scaffold was immersed in a hypotonic solution (Tris-HCl 10mM, pH 8) ON at RT and over continuous stirring at 100rpm. Then samples were treated with SDS 0.5% in the hypotonic solution for 12H (RT, 100rpm). Once treated with the detergent, samples were washed ON with the hypertonic solution (RT, 100rpm), subsequently washed three times with PBS as before for a total duration of 6H and treated with DNase I 50u/mL (Tris-HCl 10mM, pH. 7,6) and Aprotinin 10KIU/mL for 3H at 37°C under continuous stirring (100rpm) then washed 3X with PBS at RT (30min each wash). Once decellularized samples were lyophilized prior to implantation and quantitative analysis.

Powder formation: Lyophilized (L-HyC), decellularized and lyophilized (D-HyC) cartilages and collagen sponges as a control are then grinded with the CryoMill (RETSCH) in a 10 ml grinding jar of stainless steel with two 10 mm grinding balls. In order to ensure that the sample is pre-embrittled before grinding a pre-cooling time of 12 minutes is performed. Grinding is then performed for 4x 2 minutes at 25 hz and an intermediate cooling time of 30 seconds at 5Hz. Both powders are stored at 4°C for long term storage.

Biochemical analysis. After lyophilization, engineered human cartilage grafts are digested overnight at 56°C in 0.5 mL of a Proteinase K solution (Proteinase K, 1 mg/mL; pepstatin A, 10 µg/mL; 1 mM EDTA; 100 mM iodoacetamide; 50 mM Tris, all from Sigma) at pH 7.6. DNA content was evaluated fluorometrically using the CyQUANT NF Cell Proliferation Assay Kit (Thermo Fisher, USA), following the manufacturer's protocol (excitation at 485 nm, emission at 535 nm). Glycosaminoglycans (GAGs) content was analyzed by Glycosaminoglycan Assay Blyscan kit

(Biocolor) following the manufacturer instruction. Total collagen was assessed by determining hydroxyproline content after acid hydrolysis (HCL 6N, 12H, 120°C), followed by a reaction with p-dimethylaminobenzaldehyde and chloramine T (Sigma-Aldrich, USA), applying a hydroxyproline-to-collagen ratio of 0.134. Both GAG and collagen contents are normalized to the dry weight of each sample. Endotoxin quantification was performed using the Pierce™ Chromogenic Endotoxin Quant Kit (Thermo Scientific), following the manufacturer's protocol, which is based on the Limulus Amebocyte Lysate (LAL) chromogenic endpoint assay. Endotoxin quantifications are normalized to the total volume of lysate. For BMP-2 protein content, human cartilage grafts were immersed in a RIPA buffer supplemented with protease inhibitor. Tissues are then lysed with a tissue homogenizer (TissueRuptor II, Qiagen) and total BMP-2 content within tissues was assessed using the human BMP-2 DuoSet ELISA (R&D Systems) according to the manufacturer's instructions.

Cell proliferation assay. Human mesenchymal stem cells (hMSCs) from three different donors were used for all experiments. All samples were de-identified prior to use. For the proliferation assay, 1 × 10^4 cells were seeded in each well of a 24-well culture plate and allowed to attach. Following attachment, cells were treated with either collagen, L-HyC powder, or D-HyC powder at a concentration of 200 µg/mL. Cells were cultured for 5- or 7- days under standard conditions in complete media. Cell proliferation was quantified using the CyQuant assay according to the manufacturer's instructions.

Osteogenic differentiation assay. For osteogenic differentiation 5 × 10^4 MSCs were seeded in 12-well plates and cultured until reaching approximately 100% confluence (around 5 days). At confluence, cells were treated with collagen, L-HyC powder, or D-HyC powder at 200 µg/mL. Media was replaced twice weekly, with powder supplementation performed once per week. Cultures were maintained for up to 21 days under standard conditions. Three experimental groups were included: cells maintained in complete MEM medium (negative control), cells cultured in osteogenic differentiation medium (positive control), and cells cultured in complete MEM medium supplemented with the different powders. Osteogenic medium consisted of Dulbecco's Modified Eagle Medium (DMEM), high glucose supplemented with 10% fetal bovine serum (FBS), 100 U/mL penicillin and 100 µg/mL streptomycin, 50 µg/mL ascorbic acid-2-phosphate, 10 mM β-glycerophosphate and 100 nM dexamethasone.

Immunofluorescence staining. Samples were fixed in 4% paraformaldehyde (Thermo Scientific) at 4°C, for 24 h and decalcified with 10% EDTA solution (Sigma), pH 8 up to 2 weeks with gentle shaking, at 4°C. Embedding was performed using 4% low-melting agarose (Sigma) and 100 µm thick sections were cut using a 7000smz Vibratome (Campden) with stainless steel or ceramic blades (Campden). All staining and washing steps were performed under gentle shaking. Sections were blocked and permeabilized with 0.5% Triton X-100 (Sigma) in PBS and 20% donkey serum (Jackson ImmunoResearch) for 1 h, at room temperature (RT). After blocking/permeabilization, sections were stained with primary antibodies at 4°C, overnight, and highly cross-absorbed secondary antibodies for 2-3 h, at RT with 3 times 20 min washes in between using 0.1% Triton X-100 in PBS and 2% donkey serum. Tissue sections were treated with Vector TrueView Autofluorescence Quenching Kit (Vector Laboratories) to remove unwanted fluorescence. Tissue sections were mounted with a Vectashield antifade mounting medium containing DAPI (Vector Laboratories, H1200). Samples were imaged with an LSM780 confocal microscope (Zeiss). We used the following primary antibodies: mouse anti-Collagen II (Invitrogen, MA137493) and rabbit anti-Collagen Type X (COL10) (Abbexa, abx101469). The following secondary antibodies were employed: CF633 donkey anti-mouse (Sigma-Aldrich, SAB4600131) and CF568 donkey anti-rabbit (Biotium, 20098).

PBMCs preparation. Peripheral blood mononuclear cells (PBMCs) were isolated from heparinised blood from de-identified donors, using density gradient centrifugation (Lymphoprep, Axis-Shield) at 640g for 20 minutes with low break. The PBMCs were washed twice with PBS, counted, and prepared for subsequent assays. When required, monocytes were further isolated from PBMCs using CD14+ magnetic bead separation (Miltenyi) following the manufacturer's instructions, counted, and used in downstream assays. $CD4^+$ T-cells were isolated from the PBMC fraction

using the EasySep™ CD4$^+$ T-cell isolation kit (Stemcell Technologies) as per the manufacturer's instructions.

Monocyte isolation and polarization in vitro. Monocytes were isolated from freshly isolated PBMCs, as described above, from healthy controls upon informed consent (n=6, median age 43, 50% female). All samples were de-identified prior to use. Monocytes were next cultured for 5-days in 24-well plates (Falcon) at 0.5x10^{6}cells/ml in complete alpha Minimum Essential Medium supplemented with 10% heat-inactivated fetal bovine serum, 1% HEPES (1M), 1% Sodium pyruvate (100mM), 1% of Penicillin/Streptomycin/Glutamine solution and 40ng/ml M-CSF. After 5 days, media was replaced and supplemented with 200µg/ml powdered engineered human cartilage grafts (L-HyC or D-HyC) without M-CSF and cultured for an extra two days. As a control, both M1 and M2 macrophages were induced by stimulating monocytes with LPS (10 ng/mL) and IFN-γ (10 ng/mL) for M1 and IL-10 (25 ng/mL) + dexamethasone (10 nM) for M2.

Macrophage polarization analysis and preparation for T-cell proliferation. Forty-eight hour *in vitro* polarized monocytes were detached using ice-cold PBS/1mM EDTA and gentle pipetting. Next, they were washed with PBS, counted and resuspended in RPMI-1640 supplemented with 2mM L-glutamine, 10% heat-inactivated fetal bovine serum, 1% of Penicillin/Streptomycin solution. 2.5x10^3 cells were removed and used for the T-cell proliferation assay (see below). The remaining cells are washed with PBS and stained with anti-CD80, CD86 (clone FUN-1, BV650, BD), CD163 (clone: GHI/61, PE, BD) and CD206 (clone:19.2, APC/Fire750, BD), all diluted 1:100, for 20min, RT. Finally, they were washed once more with PBS and analysed by flow cytometry (CytoFLEX).

T-cell proliferation assay. T-cells isolated as described above were stained with 2µM CellTrace Violet (Invitrogen) for 20min at 37°C. Remaining dye was quenched by the addition of 6X the volume of complete medium. The cells were centrifuged, resuspended in complete medium and counted. A 96-well plate (Eppendorf) was coated with anti-CD3 (1:1000, Clone OKT3, Invitrogen) for 90min. The coating solution was removed prior to use. Wells without coating served as negative controls. In addition, anti-CD28 (clone CD28.2, Invitrogen, Thermo Fisher Scientific, 1:1000) coating was added in some wells as a positive control. Macrophages from the *in vitro* polarization assay were counted (XN-350, Sysmex) and resuspended in RPMI-1640 supplemented with 10% foetal calf serum, 2mM L-glutamine and PenStrep. Next, macrophages and T-cells at a 1:20 ratio (monocytes/macrophages: T-cells) were added to the coated plate in a total volume of 200µl. The cells were incubated for 72h, 37°C, at 5% CO_2 without media change. Subsequently, the cells were detached through gentle pipetting, centrifuged, and stained with anti-CD3 (clone UCHT1, alexa fluor 700, 1:200), anti-CD25 (clone M-A251, PerCP-Cy5.5, 1:200), anti-HLA-DR (clone G46-6, APC-H71:200) and anti-CTLA-4 (clone BNI3, PE, 1:50), all from BD, for 20min, RT. Finally, the cells were washed once with PBS and analysed using flow cytometry (CytoFLEX).

PBMCs analysis following powder activation. Previously lymphoprep isolated PBMCs were stained with 2µM CellTrace Violet (Invitrogen) as above. PBMCs were then resuspended in RPMI-1640 supplemented with 10% foetal calf serum, 2mM L-glutamine and PenStrep and culture at 0.5x10^6 in 48-well plates (Falcon). Phytohemagglutinin-M (PHA-M, 10µg/ml, Roche) was added in some wells as a positive control. PBMCs were then supplemented with 200µg/ml powdered engineered human cartilage grafts (L-HyC or D-HyC) and cultured for five days. At the end of the culture, media was collected for supplementary cytokines analysis and the cells were detached through gentle pipetting, centrifuged, and stained with anti-CD3 (clone UCHT1, alexa fluor 700, 1:200), anti-CD4 (clone RPA-T4, alexa fluor 488, 1.5:100), anti-CD8 (clone RPA-T8, PeCy7, 1.5:100), anti-CD19 (clone SJ25-C1, BV786, 1:200), anti-CD25 (clone M-A251, PerCP Cy5.5, 1:200), anti-HLA-DR (clone G46-6, APC-Cy7,1:200), all from BD, for 20min, RT. Finally, the cells were washed once with PBS and analysed using flow cytometry (CytoFLEX). IL-6, IL-10 and IFNg content within the supernatant was assessed using DuoSet ELISA (R&D systems) according to the manufacturer´s instructions.

Monocyte-derived dendritic cells (Mo-DCs) preparation and culture. Monocytes were isolated from freshly isolated PBMCs, as described above. Freshly isolated monocytes were seeded at

1x10^{6}cells/ml in 24-well plate and cultured for five days following the ImmunoCult™ Dendritic Cell Culture Kit (STEMCELL) instructions. After five days, unmatured Mo-DCs were supplemented with 200μg/ml powdered engineered human cartilage grafts (L-HyC or D-HyC), ImmunoCult™ Dendritic Cell Maturation Supplement (STEMCELL) or left untreated for 48h, 37°C, at 5% CO_2. At the end of the culture, cells were detached using ice-cold PBS/1mM EDTA and gentle pipetting. Next, they were washed with PBS, counted and stained with anti-CD14 (clone 63D3, FITC, BioLegend, 1:100), anti-CD80 (clone 2D10, BV650, 1:100, BioLegend), anti-HLA-DR (Clone G46-6, PE-Cy7, BD, 1:100).

Ectopic implantation. *FoxN1* KO BALB/C (Nude mice) & C57BL/6J (wild type) mice of 6–8-week-old were obtained from Charles River Laboratories. Nur77-GFP (Jax #016617) mice of 6–8-week-old were obtained from the Jackson laboratory. All mouse experiments and animal care were performed in accordance with the Lund University Animal Ethical Committee (M15485–18) under the regulation of the Swedish board of agriculture following the 3R's principles. Mice were housed at a 12-hour light cycle in individually ventilated cages at a positive air pressure and constant temperature. Mice were fed with rodent chow and sterile water. Immune recruitment, tissue remodeling and bone formation efficiency of engineered human cartilage grafts is assessed by ectopic subcutaneous pouches in both ATHYM-Foxn1nu/nu mice and C57BL/6J mice, with a maximum of 6 implants per animal. For surgical procedures, animals are anesthetized by inhalation using a mixture of oxygen (0,6mL min-1) and isoflurane (1.5-3vol%). After -3, -7 and-10-days or 6- and 12-weeks post implantation, samples were explanted and fixed in 4% formaldehyde solution (Solveco AB, Sweden) ON at 4°C prior to microtomography and histological analysis (Safranin-O staining) or immediately digested for FACS analysis.

Critical-sized femoral defect implantation. Ten- to 12-week-old male Sprague Dawley rats (n=15) were purchased from JANVIER LABS (France). After 7 days of acclimation the rats were anesthetized using 3% isoflurane. Then, animals were placed on a 37°C warm heating pad in prone position. Once anesthetized, isoflurane was lowered to 2 to 2.5%, and buprenorphine (Temgesic, 30 μg/kg; Indivior Europe Ltd., Dublin, Ireland) was injected subcutaneously for analgesia. The right hind limb of the animal was shaved and carefully disinfected, and an incision was made along the skin and soft tissue to expose the right femur. After cleaning the femur laterally from soft tissue, a customized in house-developed four-hole internal fixation plate (Ø of 1.5-mm straight locking plate, PEEK) was held to the lateral aspect of the femur using forceps and a clamp. The plate was then screwed to the bone using two proximal and two distal screws (Ø of 1.5-mm locking screws, stainless steel; outer screws, 7 mm in length; inner screws, 6 mm in length; DePuy Synthes). After fixation, a 5-mm osteotomy was performed using two Gigli wires (0.44 mm; RISystem AG, Landquart, Switzerland) with the help of a custom-made, three-dimensional (3D) printed saw guide. The defect was then filled with three decellularized grafts. The grafts were push-fitted and not secured with any sutures. The wound was closed in a layered fashion using resorbable sutures (Vicryl 4-0, Ethicon, Somerville, USA) by closing the muscle first (continuous interlaced suture), followed by closing the skin (Donati suture). Animals started load bearing immediately after surgery. After 6 (n=2) or 12 weeks (n=15) animals were anesthetized by isoflurane inhalation (3%), followed by CO2 asphyxiation, and right-femur were harvested prior to subsequent micro-CT, mechanical (n=7) and histological (n=3) characterization. The control empty group used in this study was part of a parallel experiment and has been previously published(18). The animals, surgical procedures, and post-operative care for this group were performed under identical conditions and protocols as the current study to ensure comparability.
All rat experiments and animal care were performed in accordance with the Swedish Board of Agriculture approval (permit number: 18-08106/2018) following the 3R's principles.

In vivo Micro-CT scanning. All rats were subjected to *in vivo* x-ray analysis at 4-6 weeks after surgery. Briefly, animals were anesthetized using isoflurane (3%) and placed in a right lateral decubitus position. Lower body was then scanned with a U-CT system (MILABS, Netherland) using a tungsten x-ray source at 50 kV and 0.21mA. A circular scan (360°) was recorded with an incremental step size of 0.250°. Volumes were reconstituted at 30μm isotropic voxel size using MILABS software analysis. For bone volume analysis, the highly mineralized tissue volume was

quantified using Seg3D (v2.2.1, NIH, NCRR, Science Computing and Imaging Institute (SCI), University of Utah, USA). For total volume analysis, each sample was meshed with Blender (v2.82a, Netherland) and analyzed with an in-house developed script.

Ex vivo Micro-CT scanning. Subcutaneous (mice) and orthotopic (rat) implanted samples were fixed overnight with 10% formaldehyde before being subjected to ex-vivo micro-CT with a U-CT system (MILABS, Netherland) using a tungsten x-ray source at 50 kV and 0.21mA for subcutaneous retrieved samples and 65KV and 0.13mA for rat-femurs. Volumes were reconstituted at 10μm isotropic voxel size and analyzed for bone volume and total volume as described in the previous section.

Mechanical testing. Femurs bearing defects and contralateral femurs were subjected to 3-point bending following previously established protocol(18). Briefly, specimens were positioned on a 3-point bending jig with 16 mm support spacing, loaded in the antero-posterior position, ensuring the defect was centred. Testing was performed using an Instron 8511.20 load frame, applying a 20 N pre-load for 10 s, followed by axial compression at 0.25 mm/s until fracture. Peak force and stiffness (linear slope) were extracted from the force-displacement curve.

Histological staining. L-HyC, D-HyC both *in vitro* or *in vivo* were washed in 1x PBS after formaldehyde fixation. Explanted *in vivo* tissues were decalcified with 10% EDTA (Sigma Aldrich, USA), pH 8.0, at 4°C for 2 weeks before paraffin-embedding. Tissues were progressively dehydrated using graded ethanol solutions (35%, 70%, 95%, and 99.5%; Solveco), with two 20-minute immersions per concentration. Following dehydration, the tissues were washed in a 1:1 mixture of 99.5% ethanol and xylene (Fisher Scientific) for 10 minutes, then treated with xylene alone for 20 minutes, twice. Subsequently, the tissues were embedded in paraffin at 56°C overnight and sectioned into 7–10 µm slices using a microtome. These sections were dried at 37°C overnight. To deparaffinize, the sections were rinsed twice in xylene for 7 minutes each, followed by a single 3-minute wash in 1:1 ethanol/xylene (99.5%). Rehydration was then performed using a graded ethanol series (99.5%, 95%, 70%, and 35%), with each step lasting 7 minutes and repeated twice.

Safranin-O staining. Sections Sections were stained with Mayer's hematoxylin solution (Sigma-Aldrich) for 10 minutes, followed by a rinse in distilled water to eliminate excess stain. Next, they were treated with 0.01% fast green solution (Fisher Scientific, USA) for 8 minutes, and any surplus dye was quickly removed by rinsing the sections in 1% acetic acid solution (Sigma Aldrich, USA) for 15 seconds. The slides were then stained with 0.1% safranin O solution (Fisher Scientific, USA) for 8 minutes. Dehydration and clearing were carried out by immersing the slides sequentially in 95% and 99.5% ethanol, followed by a 1:1 mixture of 99.5% ethanol and xylene. Finally, the sections were washed twice in xylene for 2 minutes to remove any residual ethanol and mounted using PERTEX mounting medium (PERTEX, HistoLab).

Masson´s trichrome staining. Trichrome staining was conducted using the Trichrome Staining Kit (Sigma-Aldrich) in accordance with the manufacturer's protocol. Tissue sections were deparaffinized and rinsed as previously described. The sections were then placed in Bouin's solution (Sigma-Aldrich) either overnight at room temperature or for 15 minutes at 56°C. Following this, the slides were washed under running tap water and stained with Weigert's iron hematoxylin working solution (prepared by mixing equal volumes of solution A and B, EMD Millipore) for 5 minutes to visualize nuclei (stained black). After a wash step, the cytoplasm was stained red using Biebrich Scarlet-Acid Fuchsin for 5 minutes. The slides were then cleared by immersing them in a working phosphotungstic/phosphomolybdic acid solution (25 mL phosphotungstic acid, 25 mL phosphomolybdic acid, and 50 mL distilled water) for 5 minutes. Collagen fibers were stained blue by treating the slides with aniline blue solution for 5 minutes, followed by a clearing step in 1% acetic acid solution (prepared with glacial acetic acid, Fisher Scientific) for 2 minutes and a rinse under running deionized water. Finally, sections were dehydrated by sequential immersion in graded ethanol solutions (95% once, 100% twice) for 2 minutes each, followed by two 2-minute washes in xylene, and mounted with PERTEX mounting medium.

Flow cytometry. The experiment on immune cell recruitment was carried out using flow cytometry (FACS). Under sterile conditions, harvested subcutaneously implanted samples and inguinal lymph nodes were carefully cleaned of the connective tissue. Three explanted samples from each side of the animal were pooled together for a single FACS sample and digested in an enzyme cocktail consisting of 300 U/mg collagenase type II (Sigma-Aldrich, U.S.A), 2 U/mg collagenase P (Sigma-Aldrich, U.S.A) and 2 mM $CaCl_2$ solution. The tissue digestion was carried out in a 12-well plate with 2 mL enzyme solution/well for 90 min in a humidified incubator at 37 ºC. Any tissue remnants were mechanically homogenized using a 10 mL pipette by repeatedly passing the tissue through the pipette tip. The enzymatic reaction was stopped by mixing an equal volume of cell culture medium containing 10% (v/v) heat inactivated fetal calf serum to the digest. Then the solution was passed through a 70 µM tissue strainer and collected in FACS tubes (BD, U.S.A). The cells from the digested tissues were centrifuged at 1500 rpm for 5 min, the supernatant was discarded, and the cells were resuspended in 1 mL FACS buffer (PBS 2% v/v, 2 mM EDTA). 10 ul of sample was taken and mixed with equal volume of Tryphan blue and counted using Biorad TC20™ cell counting slides (Bio-Rad, Sweden). For surface staining, cell suspension was re-spun to form a cell pellet, which was resuspended in 100 µL FACS buffer, and Fc receptors were blocked using anti-mouse CD16/32 (Clon 2.4G2, BD Biosciences) antibody for 10 min at 4 °C. Cells were then incubated with the respective primary antibodies for innate and adaptive immune system were added to the cell suspension and incubated at 4 ºC for 1 h

Antibody dilutions were added to compensation beads for setting up fluorescence compensation (UltraComp eBeads™ compensation beads, Thermofisher Scientific). At the end of incubation, cells were centrifuged, and the supernatant was discarded. Cell pellets was washed with 1 mL FACS buffer, centrifuged, and re-suspended in 350 µL FACS buffer containing nuclear stain DAPI (500 ng/ul) or Draq7 (BioLegend). The cells were then analyzed on a BD LSRFortessa™ cell analyzer (BD, U.S.A).

Antibodies list: APC-Fire 750 CD11b (M1/70, BioLegend), PE F4/80 (BM8, BioLegend), PECy7 CD11c (Bu15,BioLegend), AF647 CD206 (C068C2, BioLegend), BV650 NK1.1 (PK136,BioLegend), PerCPCy5.5 CD45 (I3/2.3, BioLegend), CD16/32 (93, BioLegend), APC Fire 750 CD8 (SK1, BioLegend), PE CD19 (1D3/CD19, BioLegend), PECy7 CD3 (17A2, BioLegend), AF647 CD4 (GK1.5, BioLegend), APC CD3 (17A2, BioLegend), BV421 CD4 (GK 1.5, BioLegend), BV510 CD8 (53-6.7, BioLegend).

Cytokines analysis. Tissue supernatants from harvested subcutaneously implanted samples on C57BL/6J or Nur77-GFP mice at day 10 post-implantation were obtained following collagenase digestion, enzyme inactivation, and centrifugation (300g, 5 min). Cytokines were quantified using LEGENDplex™ Mouse B Cell Panel w/FP (BioLegend) according to manufacturer's protocol: supernatants (1:2 diluted) were incubated with capture beads (2h), detection antibodies (1h), and Streptavidin-PE (30 min) at room temperature with continuous shaking (600 rpm). After washing, samples were analyzed on a BD LSRFortessa flow cytometer using FP (405/450 nm) and PE (532/575 nm) channels, collecting ≥300 events per bead population. Cytokine concentrations were determined using LEGENDplex™ software.

Figures

A

Protocol Number	Solution	Total nuclease incubation time (h)	Incubation rotational speed (rpm)	Total GAGs (µg/pellet)	Total DNA content (µg/pellet)
L-HyC	-	-	100	25.61 ± 2.17	12.7 ± 1.26
1	SDS 1%	-	100	9.78 ± 2.14	1.29 ± 1.10
2	SDC 1%	-	100	5.75 ± 6.27	1.57 ± 0.03
3	Triton	-	100	5.52 ± 0.51	1.47 ± 0.27
4	PBS	-	100	24.71 ± 0.52	12.2 ± 1.71
5	PBS	-	200	23.46 ± 0.39	10.94 ± 2.34
6	Hypotonic	3	-	18.28 ± 2.50	3.08 ± 0.34
7	Hypotonic	3	100	22.75 ± 0.35	1.65 ± 0.44
8	Hypotonic	3	200	6.07 ± 2.26	0.59 ± 0.07
9	Hypotonic	12	-	13.01 ± 1.61	0.21 ± 0,11
10	Hypotonic	12	100	21.65 ± 0.28	0.12 ± 0.06
11	Hypotonic	12	200	2.88 ± 0.56	0.05 ± 0.01
12	SDS 0.5%	3	100	14.83 ± 2.61	0.039 ± 0,07

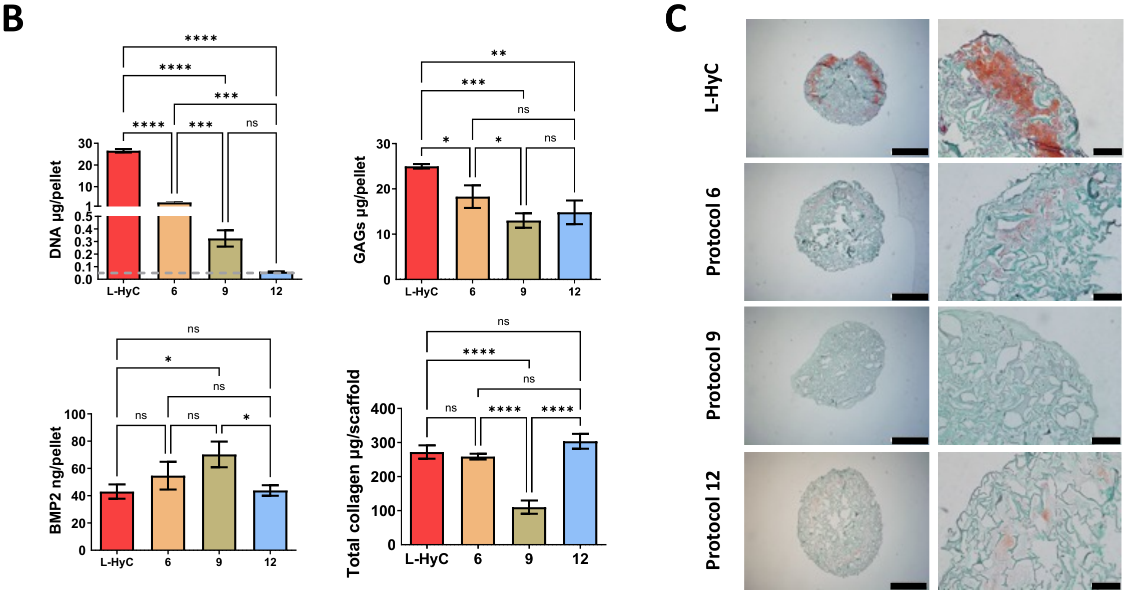

Supp. Figure 1: Development of a decellularization protocol. A. Protocol specification and resulting GAGs and DNA analysis performed on corresponding decellularized pellets (n≥3). **B.** Comparison of DNA, GAGs, Collagen and BMP-2 content on grafts after selected decellularization(n=6). Graphs represent mean ± standard deviation (SD), *p ≤ 0.05, **p ≤ 0.01, ***p ≤ 0.001, ****p ≤ 0.0001, determined by one-way ANOVA with Tukey's multiple comparisons test. **C.**Representative Safranin-O staining images of pellets exposed to the indicated decellularization protocol (scale bar= 1mm and 200µm from left to right respectively).

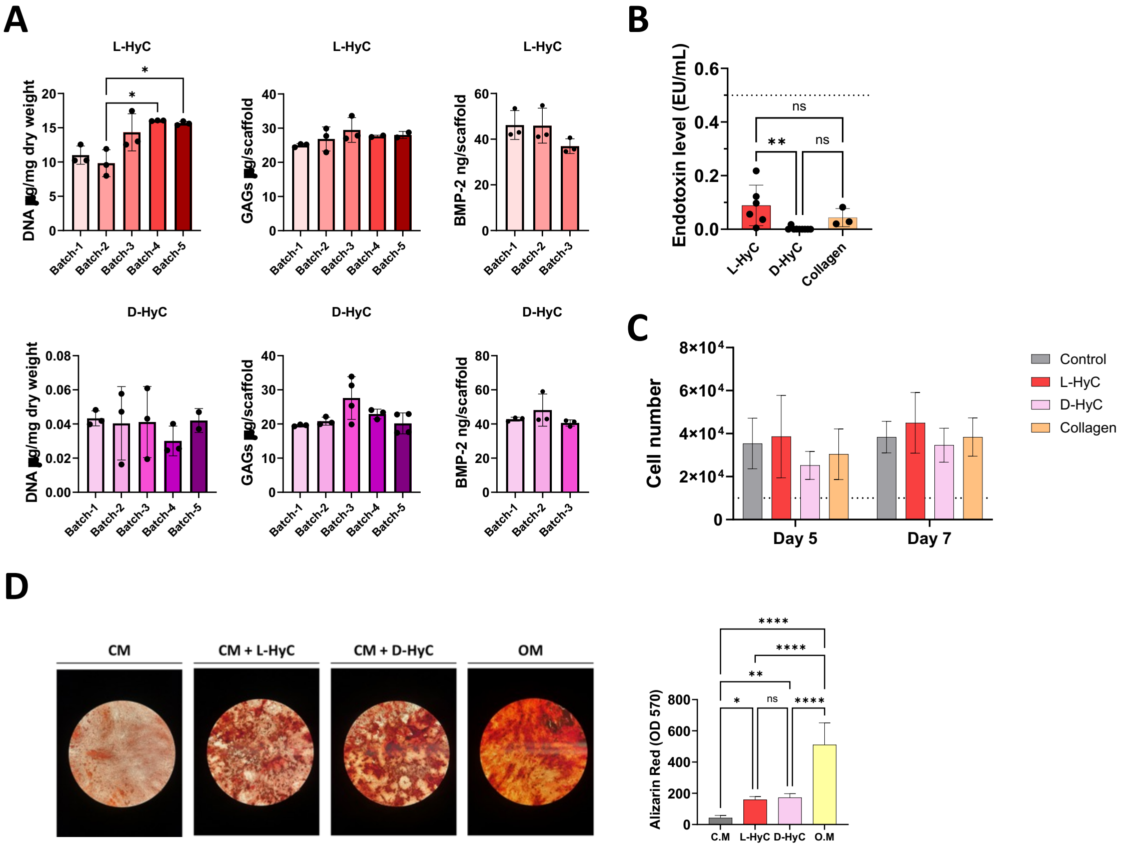

Supp. Figure 2: Standardization, reproducibility, bioactivity and safety profile of tissue engineered cartilage grafts. A. Quantification of total DNA per mg dry weight per graft, total GAGs per graft and total BMP-2 per graft. N=3 grafts were tested per batch. A standard batch generates about 100 cartilage pellets. **B.** Analysis of Endotoxins levels of both L-HyC and D-HyC. Dotted line represents the FDA threshold level defined for medical devices (0.5 EU/mL). **C.** Effect of powdered L-HyC and D-HyC on cell proliferation. Dotted line represents the initial cell seeding, 10.000 cells/well. CM: Complete Medium; OM: Osteogenic Medium. **D.** Left, Alizarin red assay on three different hMSCs donors (n=3 per donor) cultured for 14 days with powdered L-HyC and D-HyC. Right, Alizarin Red quantification.

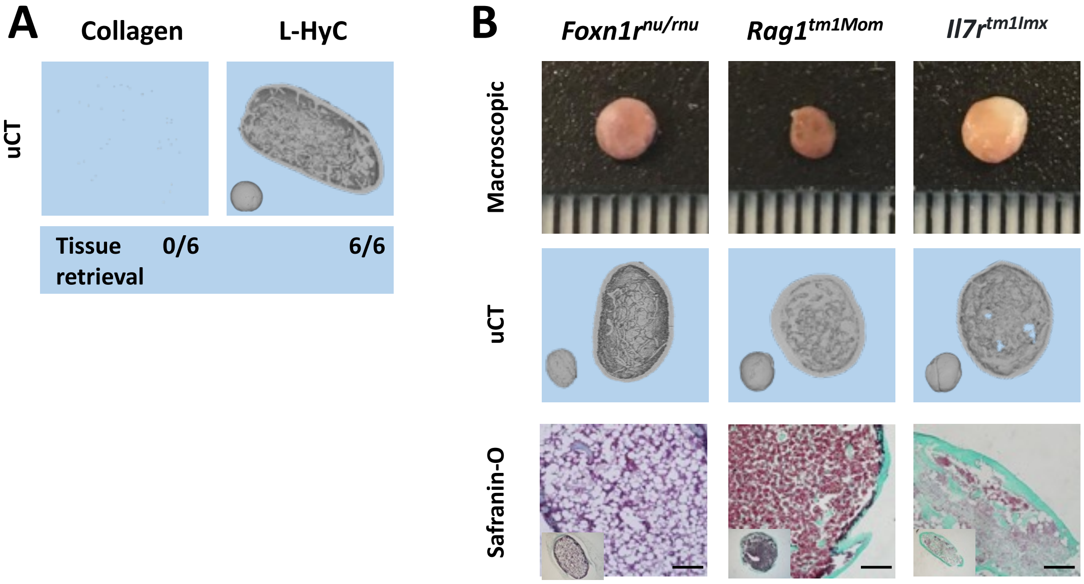

Supp. Figure 3: Bone formation relies on osteoinductive properties of L-HyC grafts. A. Representative 3D μCT images over collagen sponges (Avitene Ultrafoam) and L-HyC grafts explanted tissues at 6-weeks in Athymic Foxn1rnu/rnu mice(n=6). **B.** From top to bottom, representative macroscopic images (scale bar=1mm), representative μCT 3D reconstruction and histological images of Safranin-O of explanted tissues in different immunocompromised mice models at 6-weeks post-implantation (n=12).

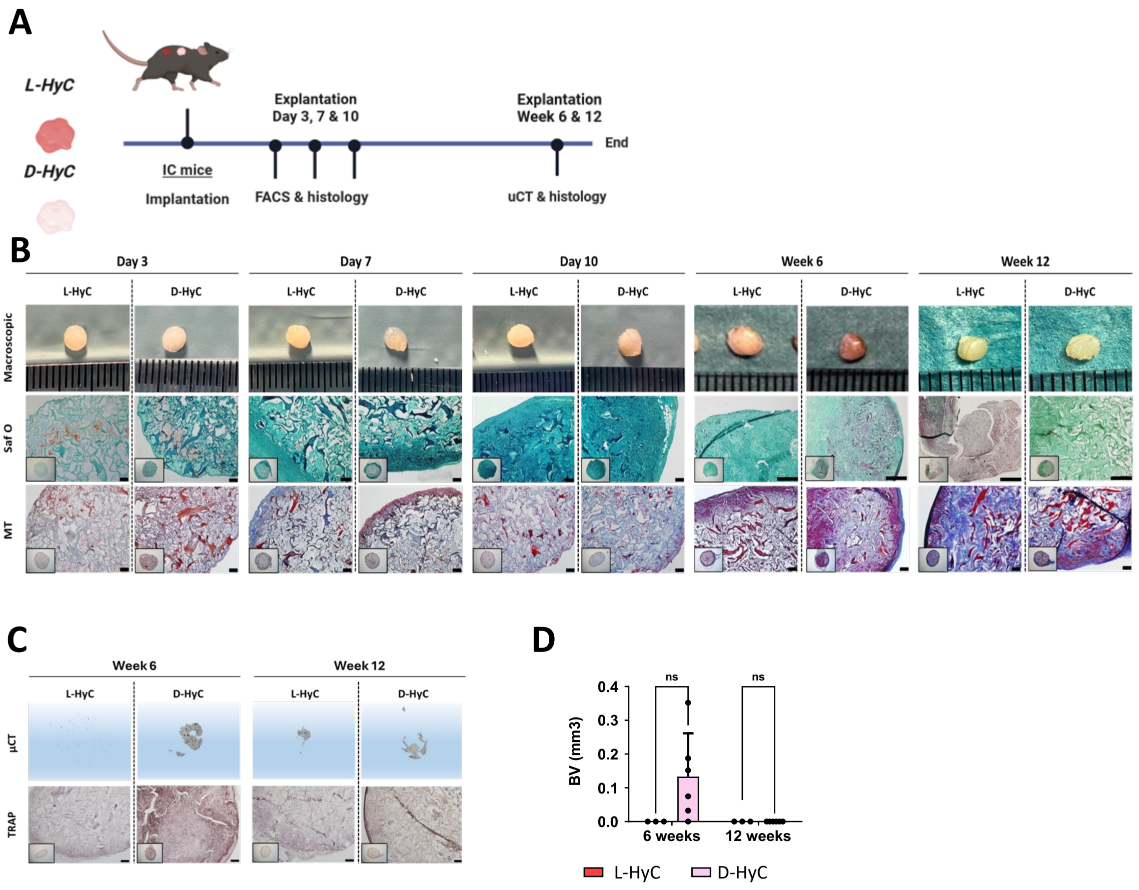

Supp. Figure 4: Decellularized human cartilage does not induce robust ectopic bone formation in immunocompetent mice. A. Experimental scheme for the in vivo ectopic osteogenic assessment of L-HyC and D-HyC grafts. **B.** From top to bottom, representative macroscopic images (scale bar=1mm), histological images of Safranin-O and Masson Trichrome staining of explanted tissues (Scale bar =100μm). **C.** From top to bottom, representative μCT 3D reconstruction and TRAP staining of explanted tissues at 6- and 12-weeks (Scale bar =400μm). In each panel, the lower image (highlighted with a black border) shows a higher magnification view of the region indicated by the black square in the corresponding upper image. **D.** BV/TV of μCT performed over explanted bones at 6- and 12-week post-implantation. The graphs represent mean ± standard deviation (SD), ns $p > 0.05$, determined by Two-way ANOVA.

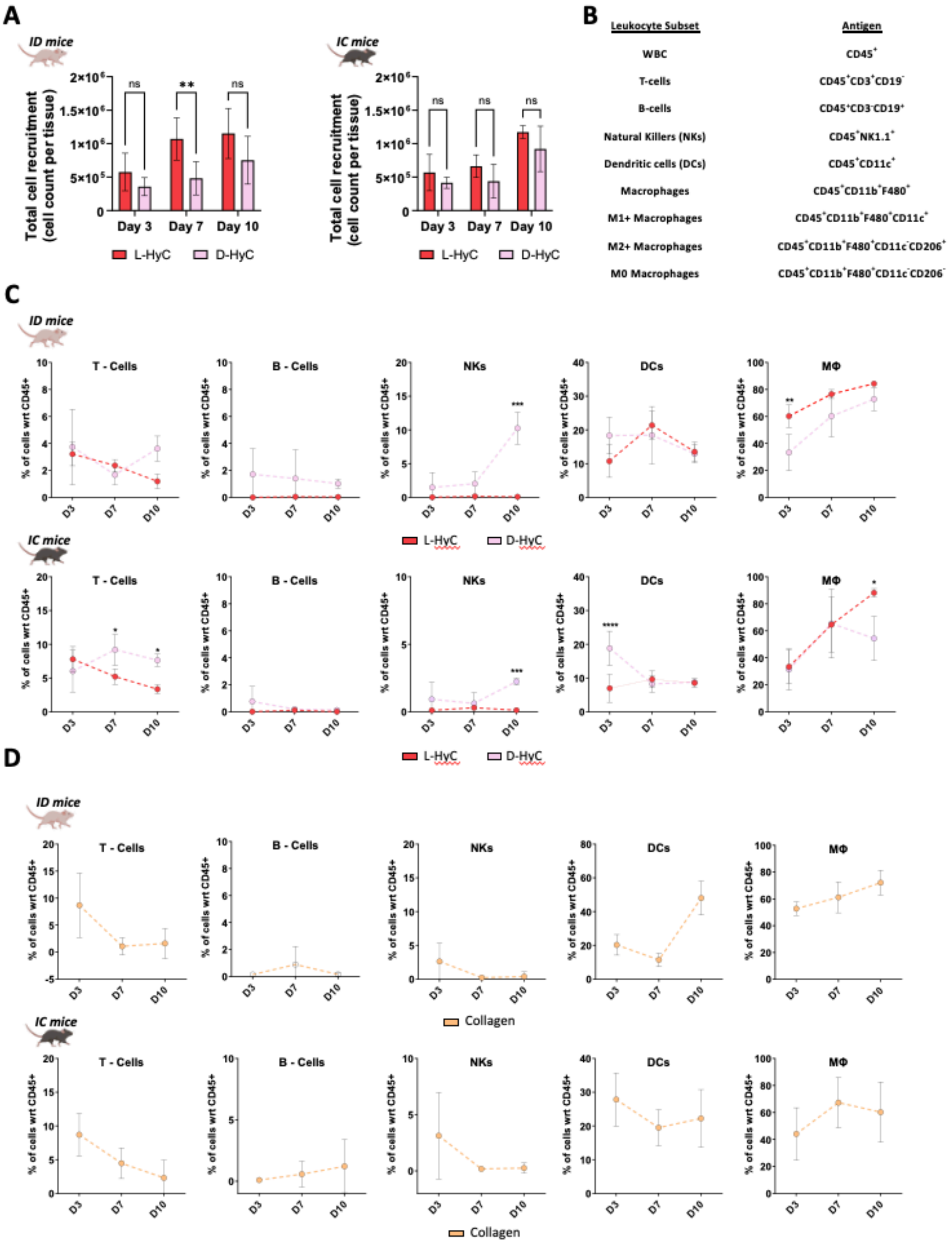
A
ID mice
IC mice
Total cell recruitment (cell count per tissue)
ns
**
Day 3
Day 7
Day 10
L-HyC
D-HyC
B
Leukocyte Subset
Antigen
WBC CD45+
T-cells CD45+CD3+CD19-
B-cells CD45+CD3-CD19+
Natural Killers (NKs) CD45+NK1.1+
Dendritic cells (DCs) CD45+CD11c+
Macrophages CD45+CD11b+F480+
M1+ Macrophages CD45+CD11b+F480+CD11c+
M2+ Macrophages CD45+CD11b+F480+CD11c-CD206+
M0 Macrophages CD45+CD11b+F480+CD11c-CD206-
C
T - Cells
B - Cells
NKs
DCs
MΦ
% of cells wrt CD45+
D3
D7
D10
D
Collagen

Supp. Figure 5: Immunogenicity assessment of engineered hypertrophic cartilage tissues in mice. A. Total cell number captured in explanted tissues after 3-, 7-, and 10-days post-implantation. The graphs represent mean ± standard deviation (SD), *p ≤ 0.05, **p ≤ 0.01, ***p ≤ 0.001, ****p ≤ 0.0001, determined by Two-way ANOVA. **B.** Flow cytometer gating strategy for T-cells, B-cells, NKs, DCs, and Macrophages. **C.** From right to left, percentages of early time recruitment of T-cells, B-cells, Natural Killers, Dendritic cells and Macrophages captured in explanted tissues after 3-, 7-, and 10-days respectively, in immunodeficient (Top) and immunocompetent (Bottom) animals. The graphs represent mean ± standard deviation (SD), *p ≤ 0.05, **p ≤ 0.01, determined by Two-way ANOVA. N≥3 independent experiments, 3 pooled samples per animal (n ≥9). **D.** From right to left, percentages of early time recruitment of T-cells, B-cells, Natural Killers, Dendritic cells and Macrophages captured in explanted collagen scaffolds after 3-, 7-, and 10-days respectively, in immunodeficient (Top) and immunocompetent (Bottom) animals. N≥3 independent experiments, 3 pooled samples per animal (n ≥9).

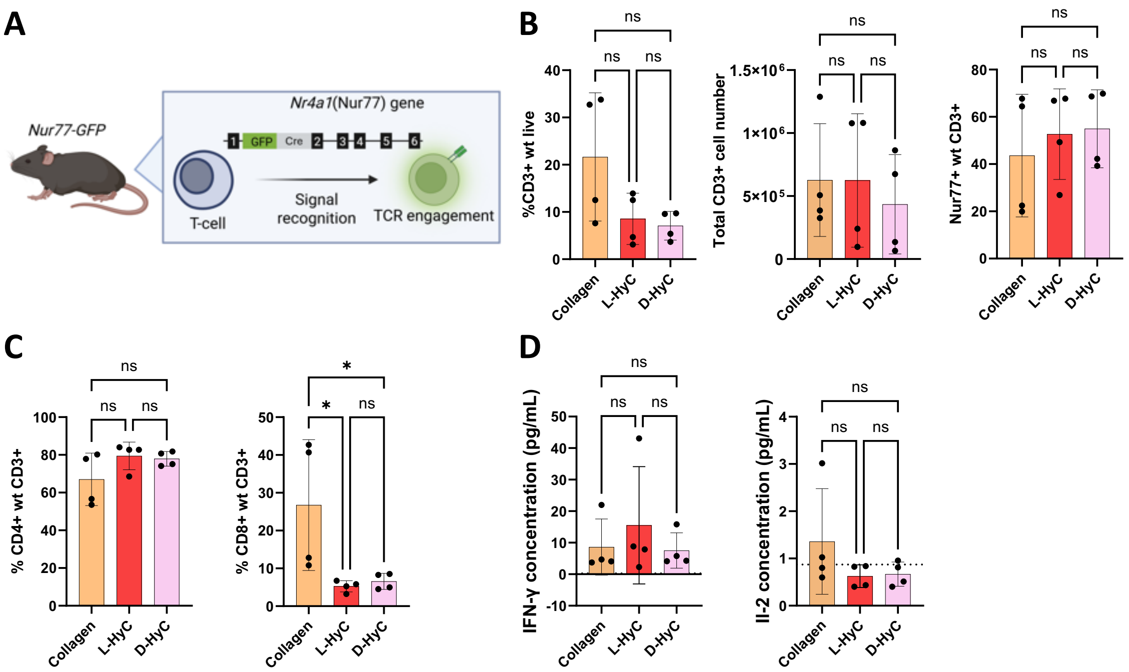

Supp. Figure 6: T-cell activation assessment of engineered hypertrophic cartilage tissues in mice. A. Nur77-GFP+ transgenic mouse expresses GFP upon TCR activation. **B.** From left to right, percentage of CD3+ cells, total CD3+ and total of activated CD3+ cells (Nur77-GFP+) captured in explanted tissues after 10-days post-implantation. The graphs represent mean ± standard deviation (SD), determined by One-way ANOVA. N≥2 independent experiments, 3 pooled samples per animal (n ≥4). **C.** From left to right, percentages of CD4+ and CD8+ T-cells out of CD3+, captured in explanted tissues after 10-days respectively. The graphs represent mean ± standard deviation (SD), determined by One-way ANOVA, $^{*}p \leq 0.05$. N≥2 independent experiments, 3 pooled samples per animal (n ≥4). **D.** Concentration of IFN-y and IL-2 recruited in in explanted tissues after 10-days post-implantation. The graphs represent mean ± standard deviation (SD), determined by One-way ANOVA. N≥2 independent experiments, 3 pooled samples per animal (n ≥4).

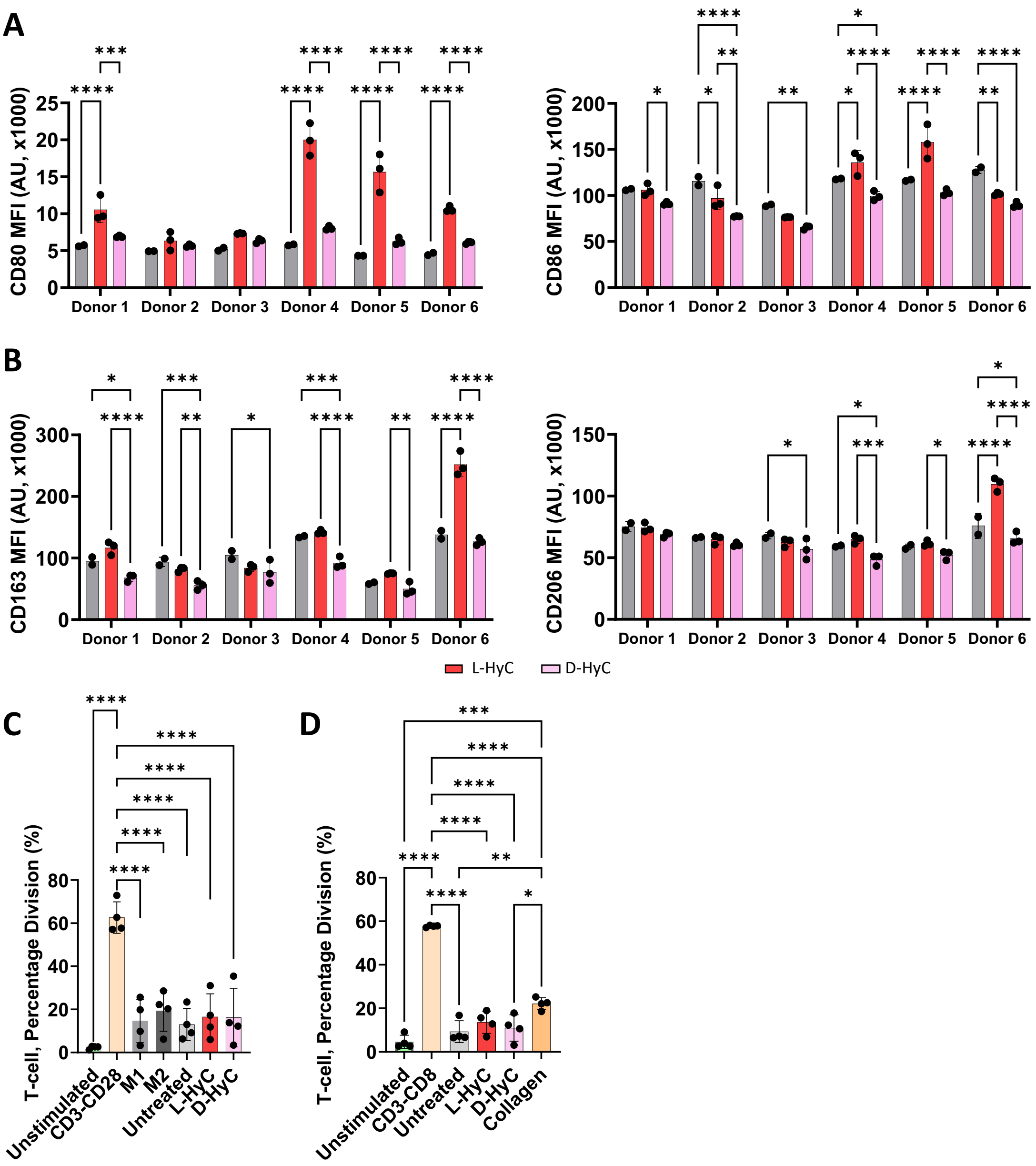

Supp. Figure 7: In vitro Immunogenic assessment of engineered hypertrophic cartilage tissues co-cultured with donor macrophages. A. Total CD80+ and CD86+ MFI indicating M1 polarization after 5-days co-culture with both L-HyC or D-HyC powdered cartilages (n=3 per donor). **B.** Total CD163+ and CD206+ MFI indicating M2 polarization after 5-days co-culture with either L-HyC or D-HyC powdered cartilages (n=3 per donor). The graphs represent mean ± standard deviation (SD), *p ≤ 0.1, **p ≤ 0.01, ***p ≤ 0.001 ****p ≤ 0.0001, determined by Two-way ANOVA. **C.** CD3+ percentage division assessed by flow cytometry after co-culture of 5x104 CD3+ cells with 2.5x103 macrophages co-cultured with either L-HyC or D-HyC powdered cartilages. The graphs represent mean ± standard deviation (SD), ***p ≤ 0.001, ****p ≤ 0.0001, determined by Ordinary one-way ANOVA (4 donors, n=3 per donor). **D.** Percentage of CD3+ division assessed by flow cytometry after co-culture of CD3+ cells (5x104 cells) with macrophages (2.5x103 cells) supplemented with either L-HyC, D-HyC or Collagen scaffold in a powdered form. The graphs

represent mean ± standard deviation (SD), determined repeated measures one-way ANOVA, statistical significance set at $p < 0,05$ (4 donors, n=3 per donor).

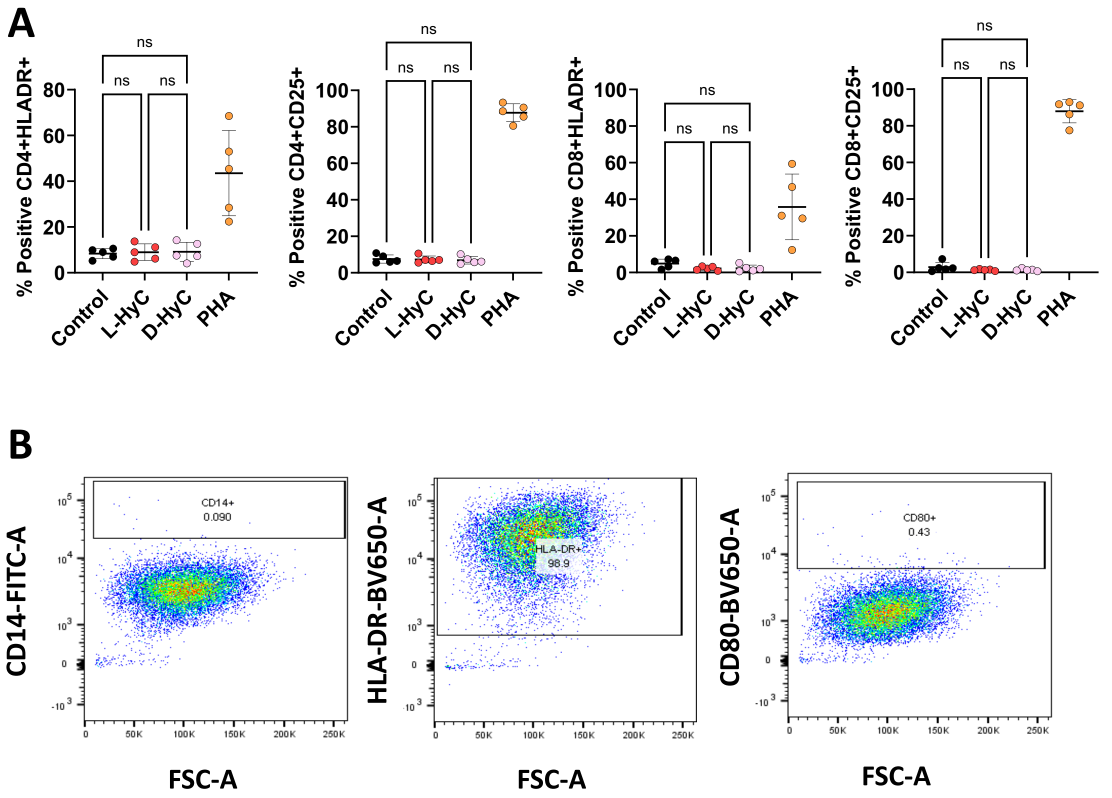

Supp. Figure 8: In vitro Immunogenic assessment of engineered hypertrophic cartilage tissues co-cultured with donor PBMCs or Mo-DCs. A. Percentage of CD3+/CD4+/HLA-DR+, CD3+/CD4+/CD25+, CD3+/CD8+/HLA-DR+ and CD3+/CD8+/CD25+ subtypes of T-cells derived from human donor PBMCs co-cultured with powdered L-HyC or D-HyC for 5-days analyzed by flow cytometry (5 donors, n=2 per donor). The graphs represent mean ± standard deviation (SD), determined by repeated measures one-way ANOVA, statistical significance set at $p < 0.05$. **B.** Representative FACS plots for monocytes derived dendritic cells (Mo-DCs) unstimulated with cartilaginous powder, indicating full dendritic differentiation (CD14-HLA-DR+) and lack of maturation (CD80+).

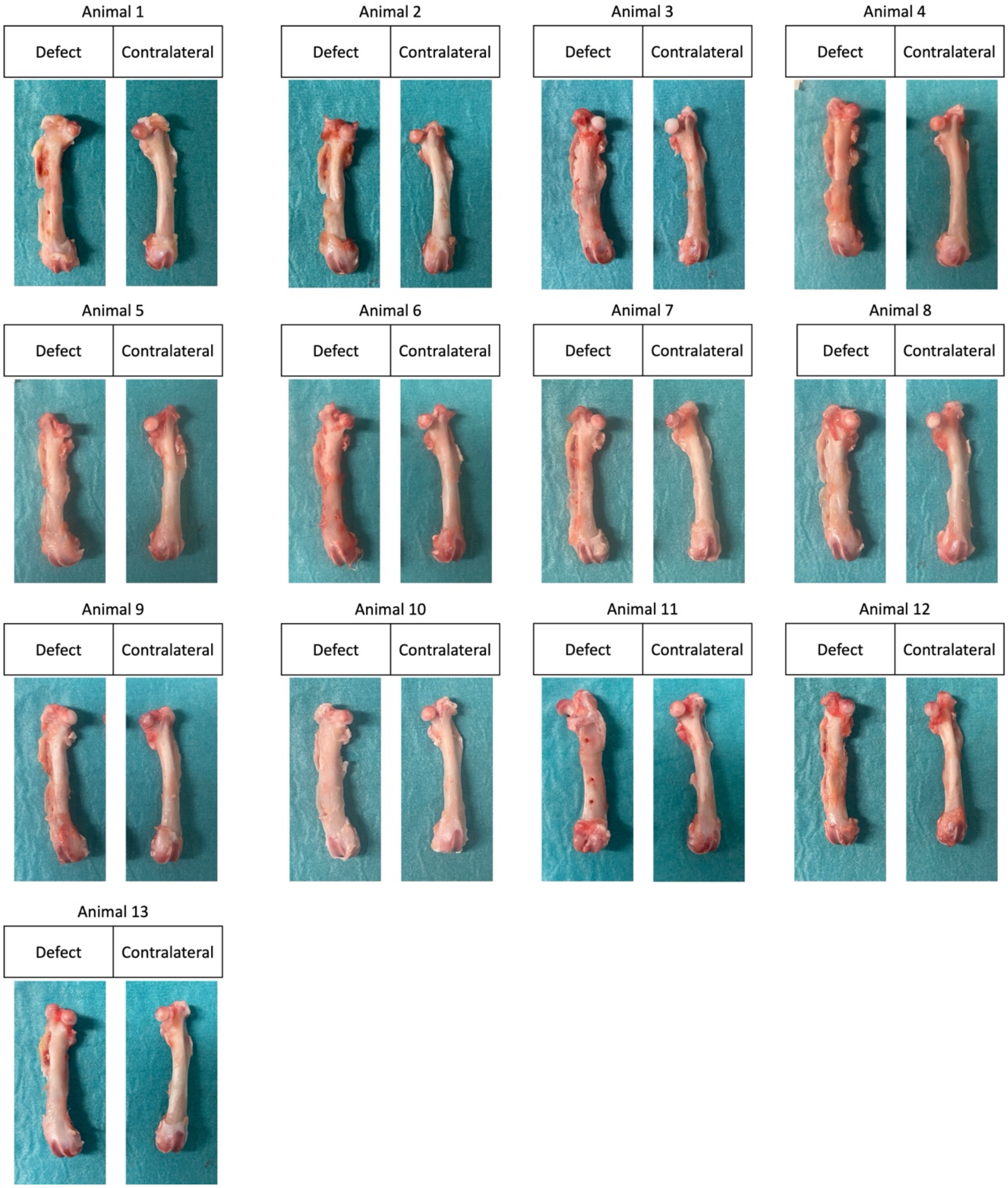

Supp. Figure 9: Macroscopic images of explanted femurs at 12 weeks post-implantation, showing the defect site (left) and contralateral control (right) for each animal.

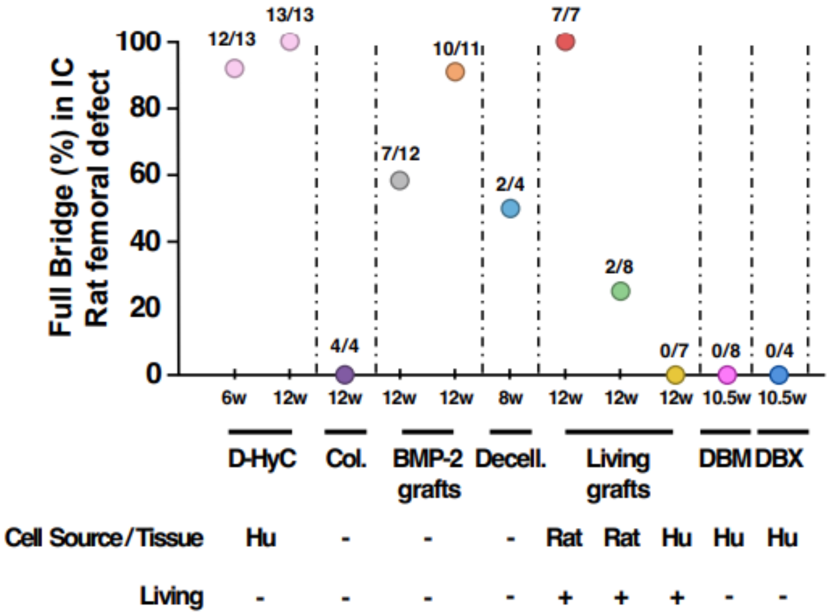

Supp. Figure 10: Performance comparison of different bone regeneration strategies in critical-sized femoral defects in immunocompetent rats. The graph shows the percentage of defects achieving full bone bridging at a specified time point for each graph (x-axis) with our D-HyC grafts (pink symbol). This comparison highlights the superior performance of D-HyC grafts, which achieved full bridging faster than all other strategies, including those requiring supraphysiological doses of BMP-2 (up to 10 µg per scaffold).

• **Collagen scaffold (Col.):** Collagen-only scaffold (purple symbol) used as a control group, data extracted from Longoni et al., 2020 (Ref. 17).

• **BMP-2 grafts:** Collagen-based scaffolds loaded with either 0.5 µg (grey symbol, left) or 10 µg BMP-2 (orange symbol, right), from adapted from Liu et al., 2023 (Ref. 18).

• **Decellularized scaffolds (Decell.):** Decellularized self-assembled human derived BM-MSC scaffolds (blue symbol) from Cunniffe et al., 2015 (Ref. 19).

• **Living grafts:** Includes syngeneic (red symbol, left), allogeneic (green symbol, middle), and xenogeneic (yellow symbol, right) living tissue engineered cartilage grafts, adapted from Longoni et al., 2020 (Ref. 16).

• **DBM:** Demineralized Bone Matrix, Grafton® DBM Putty, Medtronic. From Fassbender et al. 2014 (Ref. 20)

• **DBX: Demineralized Bone Matrix**, DBX®, Depuy Synthes. From Fassbender et al. 2014 (Ref. 20)

Cell Source/ Tissue refers to the species origin of the graft material (Hu = Human, Rat = Rat), and Living indicates whether the graft contained viable cells at implantation.

Reference	Tissue of origin	Total Wet Protocol duration (Days)	SDS Batch (Conc. & Time)	Hypotonic Media	Hypertonic Media	Nuclease (Conc. & Time)	Decellularization efficiency evaluation	Evaluation of biological performance	DNA below threshold (50ng/mg of dry tissue)
Garcia Garcia, A. et al. 2025	Human engineered hypertrophic cartilage	2.5	0.5%, 12H	Yes	Yes	50U/ml, 3H	Cell nuclei, DNA, Endotoxin levels	Yes, Osteoinductivity	Yes
(1) Ghassemi, T. et al. 2019	Bovine distal femoral joint cartilage	5	3%, 48H	No	No	No	Cell nuclei, DNA	No	No
(2) Benders, K. E. M. et al. 2014	Equine joints cartilage	4.5	N/A	Yes	No	50 U/ml, 4H	Cell nuclei	No	N/A
(3) Ching, P. C. O. et al. 2024	Porcine knee cartilage	4	N/A	Yes	No	50 U/ml, 4H	Cell nuclei, DNA	No	Yes
(4) Rahman, S. et al. 2018	Human cadaveric ears	7 to 35	N/A	No	No	2%	Cell nuclei, DNA	No	N/A
(5) Graham, M. E. et al. 2016	Human nasoseptal cartilage	3.5	N/A	Yes	Yes	90U/ml, 5H	Cell nuclei, DNA	No	Yes
(6) Browe, D. C. et al. 2019	Porcine articular cartilage	5 to 6	N/A	No	Yes	No	Cell nuclei, DNA, Endotoxin levels	No	Yes
(7) Luo, Z. et al. 2019	Porcine articular cartilage	1 to 3	1%, 24H	No	No	No	Cell nuclei, DNA	No	N/A
(8) Bautista, C. A. et al. 2016	Porcine articular cartilage	11	0.1%, 24H	Yes	No	100U/ml, N/A	Cell nuclei, DNA	No	Yes
(9) Stone, R. N. et al. 2021	Porcine articular cartilage	10	1%, 39H	Yes	No	N/A, 96H	Cell nuclei, DNA	No	Yes
(10) Luo, L. et al. 2015	Porcine articular cartilage	5 to 6	2X, 0.5%, 18H each	Yes	No	25U/ml, 16H	Cell nuclei, DNA	No	No
(11) Elder, B. D. et al. 2009	Bovine engieered cartilage	2.5	2%, 8H	Yes	Yes	0.25mg/ml, 8H	Cell nuclei, DNA	No	N/A
(12) Dos Santos et al. 2025	Bovine articular cartilage	3	1%, 48H	No	No	No	Cell nuclei, DNA	No	Yes
(13) Dortaj, H. et al. 2025	Bovine trachea	2	No	Yes	No	No	Cell nuclei, DNA	No	Yes
(14) Yang, Z. et al. 2010	Bovine articular cartilage	6.5	No	Yes	No	50U/ml, 4H	Cell nuclei, DNA	Yes, Cartilage repair	Yes
(15) Jeyakumar, V. et al. 2021	Bovine articular cartilage	2.5	No	Yes	Yes	0.0625 KU/mL, 12H	DNA	*No*	N/A

Supp. Figure 11: Overview of selected decellularization protocols applied to cartilage tissue. Studies were selected based on their similarity with our own protocol (Garcia Garcia et al.), with involvement of SDS and/or hypotonic/hypertonic washes.

References: 1. Ghassemi, T. et al. A comparison study of different decellularization treatments on bovine articular cartilage. J. Tissue Eng. Regen. Med. 13, 1861–1871 (2019).

2. Benders, K. E. M. et al. Multipotent Stromal Cells Outperform Chondrocytes on Cartilage-Derived Matrix Scaffolds. CARTILAGE 5, 221–230 (2014).

3. Ching, P. C. O. et al. Evaluation of Articular Cartilage Regeneration Properties of Decellularized Cartilage Powder/Modified Hyaluronic Acid Hydrogel Scaffolds. ACS Omega 9, 33629–33642 (2024).

4. Rahman, S., Griffin, M., Naik, A., Szarko, M. & Butler, P. E. M. Optimising the decellularization of human elastic cartilage with trypsin for future use in ear reconstruction. Sci. Rep. 8, 3097 (2018).

5. Graham, M. E., Gratzer, P. F., Bezuhly, M. & Hong, P. Development and characterization of decellularized human nasoseptal cartilage matrix for use in tissue engine ering. The Laryngoscope 126, 2226–2231 (2016).

6. Browe, D. C. et al. Glyoxal cross-linking of solubilized extracellular matrix to produce highly porous, elastic, and chondro-permissive scaffolds for orthopedic tissue engineering. J. Biomed. Mater. Res. A 107, 2222–2234 (2019).

7. Luo, Z. et al. Comparison of various reagents for preparing a decellularized porcine cartilage scaffold. Am. J. Transl. Res. 11, 1417–1427 (2019).

8. Bautista, C. A., Park, H. J., Mazur, C. M., Aaron, R. K. & Bilgen, B. Effects of Chondroitinase ABC-Mediated Proteoglycan Digestion on Decellularization and Recellularization of Articular Cartilage. PloS One 11, e0158976 (2016).

9. Stone, R. N. et al. Decellularized Porcine Cartilage Scaffold; Validation of Decellularization and Evaluation of Biomarkers of Chondrogenesis. Int. J. Mol. Sci. 22, 6241 (2021).

10. Luo, L., Eswaramoorthy, R., Mulhall, K. J. & Kelly, D. J. Decellularization of porcine articular cartilage explants and their subsequent repopulation with human chondroprogenitor cells. J. Mech. Behav. Biomed. Mater. 55, 21–31 (2015).

11. Elder, B. D., Eleswarapu, S. V. & Athanasiou, K. A. Extraction techniques for the decellularization of tissue engineered articular cartilage constructs. Biomaterials 30, 3749–3756 (2009).

12. Dos Santos, A. C. et al. From Cartilage to Matrix: Protocols for the Decellularization of Porcine Auricular Cartilage. Bioeng. Basel Switz. 12, 52 (2025).

13. Dortaj, H., Vaez, A., Hassanpour-Dehnavie, A. & Alizadeh, A. A. An update on technical method of cartilage decellularization: A physical-based protocol. BioImpacts BI 15, 30047 (2025).

14. Yang, Z. et al. Fabrication and Repair of Cartilage Defects with a Novel Acellular Cartilage Matrix Scaffold. Tissue Eng. Part C Methods 16, 865–876 (2010).

15. Jeyakumar, V. et al. Decellularized Cartilage Extracellular Matrix Incorporated Silk Fibroin Hybrid Scaffolds for Endochondral Ossification Mediated Bone Regeneration. Int. J. Mol. Sci. 22, 4055 (2021).
